# Neural Evidence for Different Types of Position Coding Strategies in Spatial Working Memory

**DOI:** 10.3389/fnhum.2022.821545

**Published:** 2022-04-20

**Authors:** Nina Purg, Martina Starc, Anka Slana Ozimič, Aleksij Kraljič, Andraž Matkovič, Grega Repovš

**Affiliations:** ^1^Department of Psychology, Faculty of Arts, University of Ljubljana, Ljubljana, Slovenia; ^2^Faculty of Medicine, University of Ljubljana, Ljubljana, Slovenia

**Keywords:** spatial working memory, fMRI, functional connectivity, encoding, maintenance, representations, prospective motor coding, retrospective sensory coding

## Abstract

Sustained neural activity during the delay phase of spatial working memory tasks is compelling evidence for the neural correlate of active storage and maintenance of spatial information, however, it does not provide insight into specific mechanisms of spatial coding. This activity may reflect a range of processes, such as maintenance of a stimulus position or a prepared motor response plan. The aim of our study was to examine neural evidence for the use of different coding strategies, depending on the characteristics and demands of a spatial working memory task. Thirty-one (20 women, 23 ± 5 years) and 44 (23 women, 21 ± 2 years) participants performed a spatial working memory task while we measured their brain activity using fMRI in two separate experiments. Participants were asked to remember the position of a briefly presented target stimulus and, after a delay period, to use a joystick to indicate either the position of the remembered target or an indicated non-matching location. The task was designed so that the predictability of the response could be manipulated independently of task difficulty and memory retrieval process. We were particularly interested in contrasting conditions in which participants (i) could use prospective coding of the motor response or (ii) had to rely on retrospective sensory information. Prospective motor coding was associated with activity in somatomotor, premotor, and motor cortices and increased integration of brain activity with and within the somatomotor network. In contrast, retrospective sensory coding was associated with increased activity in parietal regions and increased functional connectivity with and within secondary visual and dorsal attentional networks. The observed differences in activation levels, dynamics of differences over trial duration, and integration of information within and between brain networks provide compelling evidence for the use of complementary spatial working memory strategies optimized to meet task demands.

## 1. Introduction

Spatial working memory, defined as the temporary retention of spatial information when it is no longer available in the environment, is consistently characterized by sustained neural activity in a dispersed network of brain areas ranging from prefrontal and parietal cortices to posterior sensory areas (e.g., Fuster, 1973; Funahashi et al., 1993; Gottlieb and Goldberg, 1999; Zarahn et al., 1999; Brown et al., 2004; Curtis et al., 2004). This activity is thought to reflect the engagement of brain areas in active storage and maintenance processes. However, the nature of the code carried by this activity and the role of individual brain areas in maintenance of spatial information continue to be investigated. Studies have mostly focused on two possible strategies of encoding and maintenance of spatial position in spatial working memory tasks, *retrospective sensory* and *prospective motor* coding (Curtis, 2006; Funahashi, 2015). In retrospective sensory coding, the position of a target stimulus guiding motor response is assumed to be retained by spatially directed covert attention at the location of the sensory stimulus (Awh and Jonides, 2001; Postle, 2006), whereas in prospective motor coding a response motor plan is assumed to be generated at the time of stimulus presentation and maintained throughout the delay period. Experimentally distinguishing between these two strategies has proven difficult (Curtis et al., 2004; Srimal and Curtis, 2008). In this article, we present a novel paradigm that allows specific and independent manipulation of response predictability, and results suggesting that the two retention strategies are supported by separable brain systems.

Neural evidence for the distinction between retrospective sensory and prospective motor coding was first observed by single-cell recordings in prefrontal and parietal cortices of rhesus monkeys performing spatial delayed-response tasks (e.g., Niki and Watanabe, 1976; Funahashi et al., 1993; Gottlieb and Goldberg, 1999; Takeda and Funahashi, 2002), in which neuronal responses selectively represented either the position of a visual cue or the direction of a motor response. Takeda and Funahashi (2004) have shown that the selectivity of neuronal populations can change even within a single trial. Using single-cell recordings in monkey dorsolateral prefrontal cortex (DLPFC) and an anti-saccade delayed-response task that required a memory-guided saccade to be executed 90° from the target location, they observed that at the population level prefrontal neurons initially encoded the direction of the spatial position of the visual cue, and later in the trial, the direction of the required response saccade.

Similar findings have been obtained in neuroimaging studies in humans. For example, Curtis et al. (2004) used functional magnetic resonance imaging (fMRI) to reveal patterns of brain activity during performance of an oculomotor delayed-response task. In the *match* condition, participants were presented with an empty screen and had to make a memory-guided saccade to the location where the cue was previously presented, whereas in the *non-match* condition, participants were presented with two spatial cues on the screen and had to make a saccade to the cue that was not at the location of the initial cue. The authors assumed that during the *match* condition participants would be able to predict the direction of their response and would therefore rely on prospective motor coding of spatial locations, whereas in the *non-match* condition they would not be able to predict the direction of the response and would have to rely on retrospective sensory coding. Their results showed higher delay-period activity in the frontal eye fields (FEF), supplementary eye fields (SEF), and middle frontal gyrus (MFG) for the *match* condition and in the intraparietal sulcus (IPS) for the *non-match* condition, suggesting different contributions of brain areas to the hypothesized coding mechanisms.

There were, however, a number of limitations in the Curtis et al. (2004) study that affect the validity of the authors' conclusions and require their further investigation. Importantly, the two task conditions contrasted in the study were not matched in difficulty, required precision of representation, and specific memory retrieval processes (Srimal and Curtis, 2008). Whereas, the *match* condition required maintenance of the precise target position and its successful recall, correct performance in the *non-match* condition required only a coarse representation of the target position and its successful recognition. A subsequent study by Srimal and Curtis (2008) used a similar spatial delayed-response task in which the memory-guided saccade and spatial item-recognition task conditions were matched in difficulty by an adaptive psychophysical procedure that matched the spatial precision required in the item-recognition condition to performance in the memory-guided saccade condition, separately for each participant. This time, comparison of task conditions revealed no differences in delay-period activity as measured with fMRI, leading the authors to conclude that the observed FEF activity may reflect maintenance of a spatial map rather than a saccade motor plan.

Although, the Srimal and Curtis (2008) study succeeded in holding task difficulty constant across conditions, some important limitations and open questions remain. The authors purposefully kept the two task conditions different in terms of the type of response required—one required a saccade, the other a perceptual judgement communicated by a button press—to avoid FEF activity in the anticipation of an eye movement in the item-recognition task condition that did not promote prospective motor coding. Although planning an eye saccade was not necessary for task performance, it was not directly hindered by the task or incompatible with successful task performance. It could easily have been used as a supporting strategy in the item-recognition condition. Similarly, retrospective sensory coding is also a viable supporting strategy in the memory-guided saccade condition. In summary, the task design may not have elicited significant differences in task performance strategies and associated representations between the two conditions, so these could not be robustly identified.

The aim of our study was to advance the exploration of neural bases of position coding in spatial working memory. Our goal was to investigate whether different spatial working memory strategies and related representations are used depending on specific task demands and to identify their neural correlates. Similar to Curtis et al. (2004) and Srimal and Curtis (2008), we focused on comparing conditions that allowed prospective planning and maintenance of a motor plan with conditions in which the motor response could not be predicted and participants could therefore rely only on retrospective sensory information. To overcome the limitations of previous studies, we designed a task that, first, matched not only the task difficulty across the conditions, but also the type of memory retrieval (recall vs. recognition) and the required response, and, second, actively discouraged prospective motor coding, because it would interfere with successful performance in the condition that favored retrospective sensory coding. To the best of our knowledge, this is the first study of spatial working memory in which the predictability of the motor response was independently manipulated. This allowed us to compare sensory and motor coding of precise spatial position without task difficulty, response type, or type of memory retrieval (recall vs. recognition) confounds. Whereas, previous studies have focused primarily on patterns of brain activity, we also explored the possibility that a difference in task strategies used would lead to a difference in the flow of information between different brain regions and networks, which in turn would lead to observable differences in functional connectivity patterns.

We report the results of two separate experiments. In the first experiment (Experiment I), we used a full factorial design with independent manipulation of response predictability and task difficulty. In the second experiment (Experiment II), we focused on response predictability in conditions of precise spatial recall only. In both experiments, we expected that retrospective sensory coding would be reflected in increased activity and connectivity in parietal and posterior sensory areas, whereas prospective motor coding would be associated with increased activity and connectivity in frontal areas involved in motor planning and execution. In addition, we expected that differences in strategies would be time-dependent, with earlier phases of the delay period reflecting preparation of a prospective motor plan and later phases of the delay period reflecting maintenance and reactivation of retrospective sensory information.

## 2. Materials and Methods

### 2.1. Participants

Thirty-one (20 women, 23±5 years) and 44 (23 women, 21±2 years) participants took part in two spatial working memory experiments. The number of participants was selected to exceed sample sizes of comparable fMRI studies on spatial working memory (e.g., Curtis et al., 2004; Srimal and Curtis, 2008) and was sufficient to detect medium effects of interest (*d* = 0.5) at *p* < 0.05 with 0.8 and 0.9 power in Experiments I and II, respectively. All participants were healthy young adults with no current or previous neurological, psychiatric, or substance use disorders. Exclusion criteria also included any contraindications to MR or EEG, such as the presence of metal implants or other metal particles in the body, a history of epileptic seizures, tremor, or other motor disorders, and pregnancy. All participants had normal or corrected-to-normal vision. Participants performed the task with their dominant hand, with the majority of participants being right-handed and only two participants being left-handed (Experiment I). Due to the small number of left-handed participants, we decided to exclude these participants to ensure a homogeneous sample. In addition, we excluded 13 participants due to incomplete data collection and poor data quality (Experiment I: 6 participants, Experiment II: 7 participants). Demographics of participants included in the data analysis can be found in Table 1. Both experiments were approved by the Ethics Committee of the Faculty of Arts, University of Ljubljana, Slovenia, while the second experiment was additionally approved by the National Medical Ethics Committee of the Ministry of Health of the Republic of Slovenia. All participants gave written informed consent prior to participating in the study.

**Table 1:** Demographic data of participants included in data analysis.

**Experiment**	**Number**		**Age (years)**				**Handedness**
	**All**	**Females**	**Range**	**All (SD)**	**Females (SD)**	**Males (SD)**	**Right**
I	23	15	18–38^*^	23.77 (5.97)^*^	21.21 (3.04)^*^	28.25 (7.32)	23
II	37	20	19–30	21.00 (2.57)	19.80 (1.20)	22.41 (3.04)	37

* *Missing information for one participant*.

### 2.2. Behavioral Task

#### 2.2.1. Experiment I

**Figure 1:**
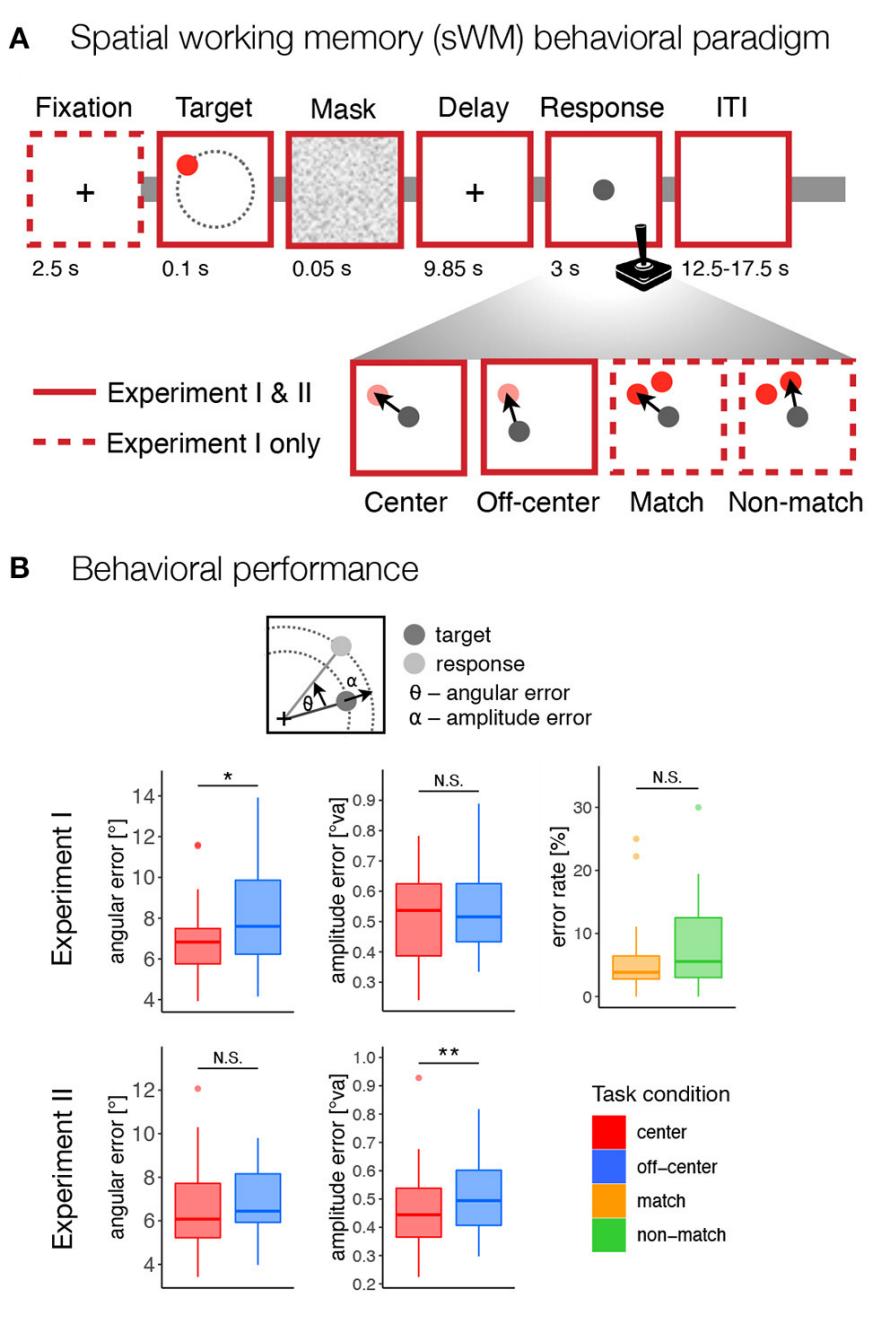
Behavioral paradigm and performance. **(A)** The design of the spatial working memory tasks in both experiments. Participants were asked to remember the position of a briefly presented target stimulus (red disk) and, after a delay period, to move a gray disk using a joystick to the position of the remembered target or an indicated non-matching location. The task consisted of four conditions: *center*–move to target position from the center of the screen, *off-center*–move to target position from a random off-center position, *match*–move to the position of the probe at the target position, *non-match*–move to the position of the probe that does not match the target position. **(B)** Behavioral results for both experiments. For the *match* and *non-match* conditions, the response error was calculated as the percentage of incorrect responses. For the *center* and *off-center* conditions, the response error was calculated as the standard deviation of the angular and amplitude errors between the target and response positions, corrected for systematic biases. N.S., not significant; *p < 0.05, **p < 0.01.

Participants in both experiments performed a delayed-response spatial working memory task (Figure 1A). In Experiment I, each trial began with the presentation of a fixation cross in the center of the screen for 2.5 s. Participants were instructed to keep their gaze at the center of the screen throughout the trial until the response. Next, a target stimulus (red disk with a radius of 50 px, 0.531°va) was presented for 0.1 s on the screen. Across trials the targets were shown at locations with a constant radius (400 px, 4.24°va) and at different angles from the center of the screen (36 unique positions from 5° to 355° in 10° increments), followed by a brief 0.05 s masking pattern used to disrupt iconic visual memory (Curtis et al., 2004). The target stimuli were never presented at the cardinal axes to prevent verbalization of the precise positions (Srimal and Curtis, 2008). Participants were asked to remember the position of the target stimulus and to maintain it during the following 9.85 s delay period. After the delay period, participants responded by moving a gray disk (50 px, 0.531°va radius) to the position of the remembered target or to an indicated non-matching location using a joystick. The response time was fixed, such that the position of the gray disk after 3 s was recorded as the response position. Individual trials were separated by inter-trial intervals (ITI) whose duration varied randomly (12.5, 15, or 17.5 s with a ratio of 3:2:1) to allow for better task-related BOLD signal decomposition.

The task consisted of four conditions—*center, off-center, match*, and *non-match*—that differed in the required precision of encoded spatial representations and the possibility of preparing a motor response at the time of stimulus presentation and working memory encoding. In the *center* and *off-center* conditions, the gray disk had to be moved to the target position on an empty screen. In the *center* condition, the gray response disk always appeared in the center of the screen. In the *off-center* condition, the gray response disk always appeared off-center with a constant radius (400 px, 4.24°va) but at a random angle to the target position, excluding any locations that were too close to the screen edges (<100 px, 1.06°va, between the edge of the gray disk and the screen edges). In the *match* and *non-match* conditions, two red disks (each with a radius of 50 px, 0.531°va) appeared on the screen during the response period. One of the disks appeared at the position that matched the target position, while the other disk appeared at the same amplitude from the center 50° (30° for only one participant) clockwise or counterclockwise from the target position. The position of the non-match target varied in a pseudorandom manner and was counterbalanced across the trials. The gray response disk was presented at the same time in the center of the screen. In the *match* condition, the response disk had to be placed over the red disk at the position that matched the target. In the *non-match* condition, participants had to place the response disk over the red disk at the position that did not match the target position.

In summary, the task design required participants in the *center* and *off-center* conditions to encode and recall spatial information using a high-precision representation, whereas in the *match* and *non-match* conditions they could rely on coarser spatial encoding and recognition that allowed match-to-sample responses. In this way, we were able to contrast conditions with different representation requirements at different task difficulty levels. In the *off-center* and *non-match* conditions, the required motor response was not known prior to the onset of response period, since the response either began or ended at random, unpredictable locations, respectively. Importantly, the prospective motor coding in these two conditions was not only uninformative but also misleading, as it interfered with the actual motor response that participants were required to make. We hypothesized that this would discourage participants from creating a prospective motor plan and instead encourage them to rely solely on retrospective sensory representations. The reverse, however, was not true in the *center* and *match* conditions. In the *center* and *match* conditions, the required motor response was known at the time of target presentation, allowing participants to complete the task by using either retrospective sensory coding, prospective motor coding or both coding strategies.

The data in Experiment I was collected in a single session. Participants first performed a short practice to familiarize themselves with the task and the use of joystick. During the practice, we described the task conditions and the required responses. Participants then performed the task while their brain activity was observed using fMRI recording. The task was performed across eight scanning runs. To enable and encourage the use of a stable strategy, each run included only one task condition (Curtis et al., 2004). Participants were informed about the task condition and reminded about the instructions immediately before the beginning of each task run. The runs were performed in a fixed order, specifically: *center, match, non-match, off-center, off-center, non-match, match*, and *center*. The order was chosen to counterbalance the effects of training and fatigue across task conditions and to group responses of the same type (free recall vs. match-to-sample). Each run consisted of 18 trials, yielding 36 trials per condition and 144 trials in total.

#### 2.2.2. Experiment II

The task in the second experiment introduced two modifications. First, the task did not include the initial fixation period, but began directly with the presentation of the target stimulus. The onset of the fixation cross in Experiment I alerted participants to the upcoming trial. This was beneficial in preparing participants to efficiently encode a briefly presented stimulus. However, the fixation cross presented a sensory event in itself and elicited an orienting response. Although the perceptual and orienting response should be stable across task conditions and should not interfere with their direct comparison, we decided to omit it in Experiment II to simplify the trial event structure and the assumed modeling of the BOLD signal. Second, as our primary interest was in comparison of neural correlates of high-precision spatial working memory representations, we omitted the *match* and *non-match* conditions.

In this experiment, the timing and duration of various phases of a task trial remained the same as in the first experiment with the exception of the ITI durations, which were varied pseudorandomly between 15, 16, or 17 s with a ratio of 3:2:1. The target stimulus (red disk, radius 50 or 25 px, depending on screen resolution, and 0.531 or 0.351°va, depending on viewing distance—the smaller stimuli were presented to two participants only) was presented at 24 different positions from 7.5 to 352.5° in 15° increments. In the *center* condition, the gray response disk appeared in the center of the screen. In the *off-center* condition, the gray disk appeared at a constant amplitude (400 or 200 px, 4.24 or 2.81°va) from the target position but at randomly varying angles from the target position, excluding positions that were too close to the screen edges (less than 100 or 50 px, 1.06 or 0.702°va, between the edge of the gray response disk and the screen edges).

The data for Experiment II was collected over one to three sessions (6 participants with one session, 12 participants with two sessions, and 19 participants with three sessions). On each individual session, after training, the participants performed the task in two separate runs during which concurrent fMRI and EEG signal was recorded. Each run consisted of 24 trials of the same task condition, resulting in 24, 48, and 72 trials per condition, 48, 96, and 144 trials in total, for one, two, or three sessions, respectively. In the first session, the order of task conditions was pseudorandomly varied and counterbalanced across participants. In each subsequent session with the same participant, the order was reversed from the previous session. Data from participants who attended multiple sessions were combined across sessions. In this article, we report results based only on behavioral and fMRI data. While EEG data are not the focus of this paper, we used EOG signal to identify eye movements during task performance.

#### 2.2.3. Control for Eye Movements

In Experiment II, we used the electrooculography (EOG) signal extracted from the EEG data to check whether participants followed instructions and avoided eye movements during the presentation of the target and the delay phase of the spatial working memory task. Details of EEG data acquisition and EOG analysis can be found in the Supplementary Material. Briefly, the results of the EOG analysis showed that participants did not move their eyes on more than 7.81% (median, IQR = 15.8%) of trials during the *center* condition and 11.1% (median, IQR = 16.7%) of trials during the *off-center* condition. Using logistic regression to test for differences between task conditions, we found no evidence of systematic differences in saccade frequency between task conditions (β = 0.258, *Z* = 1.87, *p* = 0.061, *OR* = 1.29).

#### 2.2.4. Materials

In both experiments, stimuli were displayed on an MR-compatible screen (Esys Patient Display, *Invivo*, 2012, monitor size: 640 × 400 mm) that was visible to participants from the MR scanner *via* a head mirror (Experiment I—screen resolution: 2, 560 × 1, 600 px, viewing distance: 1, 350 mm; Experiment II—screen resolution: 2, 560 × 1, 600 or 1, 280 × 800 px, viewing distance: 1, 350 or 2, 040 mm). The task was prepared using a custom Python script and the PsychoPy software (Peirce, 2007). Participants' responses were collected using an MR-compatible joystick (Hybridmojo LLC, Washington, USA).

### 2.3. Behavioral Data Analysis

To standardize the behavioral measurements across different screen resolutions and viewing distances, we first converted the pixel-based measurements to degrees of visual angle. We then excluded all invalid responses and outliers to ensure that the results reflected the engagement of spatial working memory, rather than technical errors or inattention to the task. For the *match* and *non-match* conditions, any response that did not overlap with the matching or non-matching stimulus was marked as an incorrect response, respectively (6.88% of excluded trials). For the *center* and *off-center* conditions, we used several criteria to identify outliers. First, any response that was more than 45° from the target position in either direction or whose amplitude was not between 0.5 and 1.75× target amplitude was excluded from further analysis. Due to the potentially large impact of outliers, we additionally excluded all responses that fell outside the 1.5× IQR boundaries for either angular or amplitude error (a total of 10.6% of excluded trials in Experiment I and 8.02% of excluded trials in Experiment II).

Next, we examined task performance in each task condition. For all conditions, we used the final position of the response disk as the participant's response. For *match* and *non-match* conditions, we computed error rates as the proportion of trials in which participants did not correctly place the response disk to overlap the red disk at the position that matched the target or the red disk at the alternate position, for *match* and *non-match* conditions, respectively. We tested the difference in error rates between *match* and *non-match* conditions by computing a logistic regression model with the task condition as a predictor and testing its statistical significance with a *Z*-test.

In *center* and *off-center* conditions, which required precise reconstruction of the target position, we used the final position of the response target relative to the target position to assess the precision of the behavioral responses. As findings from single-neuron recordings suggest that at the neuronal level spatial representations are encoded in terms of an angle and an amplitude, in other words in the polar coordinate system (Funahashi et al., 1989; Chafee and Goldman-Rakic, 1998; Rainer et al., 1998), we decomposed the response error into angular and amplitude differences between the target and response positions measured from the center of the screen (Figure 1B). We used the center of the screen as a point of origin as that is where participants had to keep their gaze during target presentation and delay periods and thus it served as the best origin point for working memory representation of target location. In addition, a number of experiments (Huttenlocher et al., 1991, 2004; Haun et al., 2005) and previous findings from our group (Starc et al., 2017) have suggested the presence of systematic effects on behavioral responses, such as systematic under- or overshooting of responses and a tendency to shift responses closer to the nearest diagonals. Because these systematic biases can lead to misestimation of trial-to-trial precision error and summary precision measures, we estimated the magnitude of systematic biases using a modified procedure described in Starc et al. (2017). Specifically, we calculated the systematic amplitude bias as the average amplitude error, with negative values reflecting an undershoot and positive values reflecting an overshoot. We calculated the systematic angular bias as the average angular error at each distinct angle from the nearest diagonal, with positive values reflecting a response bias toward the diagonal and negative values reflecting a response bias away from the diagonal. We estimated the systematic biases for each participant separately and then subtracted them from the original recorded trial-by-trial responses to obtain corrected angular and amplitude errors. We then calculated the standard deviation of the corrected angular and amplitude errors across all trials of the same condition as a measure of response spread and to serve as an overall estimate of precision errors for each individual.

We compared precision errors and systematic biases between task conditions using permutation analysis (10, 000 permutations) and paired *t*-tests in PALM (Winkler et al., 2014). We adjusted the observed *p*-values for multiple comparisons using the FDR correction. The remaining data analysis and visualizations were performed in R (R Core Team, 2021). We also calculated effect sizes between statistically significant comparisons using Cohen's d measure.

### 2.4. fMRI Acquisition, Preprocessing, and Analysis

In both experiments, MRI data were collected with Philips Achieva 3.0T TX scanner. T1- and T2-weighted structural images were acquired for each participant (Experiment I—T1 and T2: field of view = 224 × 235 mm, 236 sagittal slices, matrix = 320 × 336, voxel size = 0.7 × 0.7 × 0.7 mm; T1: TE = 5.7 ms, TR = 12 ms, flip angle = 8°; T2: TE = 414 ms, TR = 2, 500 ms, flip angle = 90°; Experiment II—T1 and T2: field of view = 224 × 235 mm, 236 sagittal slices, matrix = 320 × 336, voxel size = 0.7 × 0.7 × 0.7 mm; T1: TE = 5.8 ms, TR = 12 ms, flip angle = 8°; T2: TE = 394 ms, TR = 2, 500 ms, flip angle = 90°). We recorded brain activity using BOLD images with T2*-weighted echo-planar imaging sequence (Experiment I—8 BOLD images, field of view = 240 × 240 mm, 48 axial slices, voxel size = 3 × 3 × 3 mm, matrix = 80 × 80, TR = 2, 500 ms, TE = 27 ms, flip angle = 90°, SENSE factor 2, 215 frames; Experiment II—2-6 BOLD images, field of view = 240 × 240 mm, 56 axial slices, voxel size = 2.5 × 2.5 × 2.5 mm, matrix = 96 × 95, TR = 1, 000 ms, TE = 48 ms, flip angle = 62°, MultiBand SENSE factor 8, 698 frames).

MRI data were preprocessed and analyzed using Quantitative Neuroimaging Environment and ToolboX (QuNex; Ji et al., in preparation). MR images were preprocessed using the Human Connectome Project (HCP) minimal preprocessing pipeline (Glasser et al., 2013). Specifically, the structural images were corrected for magnetic field distortions and registered to the MNI atlas, brain tissue was segmented into white and gray matter, and the cortical surface was reconstructed. Functional BOLD images were slice-time aligned, corrected for spatial distortions, motion corrected, registered to the MNI atlas, and the BOLD signal was mapped to a common surface volume (CIFTI) representation and spatially smoothed (σ = 4). Further analyses were performed on “dense” whole-brain data (i.e., each voxel and vertex independently). To increase statistical power and to observe general patterns of task activations and differences across identified functional brain parcels, we also performed analyses on parcellated whole-brain data. Parcellated data were obtained by extracting the mean signal for each region of interest (ROI) as identified in the HCP-MMP1.0 parcellation (Glasser et al., 2016).

We performed the activation analysis using a GLM approach in which event regressors were convolved with the assumed SPM hemodynamic response function (HRF; Friston et al., 1994). The task employed a slow-event related study design with longer ITIs (12.5–17.5 s) that should ensure the BOLD signal to return to baseline before each new trial. This ensured that baseline levels were stable and comparable across task conditions performed within separate BOLD runs. For each participant, we separately modeled each phase of the trial for each task condition. Specifically, we estimated β coefficients for the trial onset (Experiment I: −2.5 to 0.15 s, Experiment II: 0–0.15 s), delay (0.15–10 s), and response (10–13 s) phases of a trial for each task condition for both dense and parcellated data (Supplementary Figure 2A). To identify possible temporal changes during the delay period, in a separate analysis, we additionally modeled the delay phase with separate regressors for early (0.15–5 s) and late delay (5–10 s), focusing only on whole-brain data (Supplementary Figure 2A). Trials with outlier responses based on behavioral data analysis were modeled as separate events and excluded from the group-level statistical analyses of the fMRI data. In Experiment I, an average of 33 out of a total of 36 trials per task condition were included in the analysis. In Experiment II, an average of 22 of 24 trials, 45 of 48 trials, and 67 of 72 trials per task condition were included in the analysis for participants that attended only one, two, and three sessions, respectively. Baseline was estimated separately for each BOLD run.

We then analyzed the β estimates at the group level using permutation analysis (500 permutations, tail acceleration). To identify activation and deactivation during individual events based on the dense whole-brain data, we performed two-tailed one-sample *t*-tests with cluster (*C* = 3.1) FWE correction. To identify regions activated across pairs of conditions, we performed a conjunction analysis (Heller et al., 2007) to compute pooled *p*-values. The resulting images included grayordinates that exhibited activation or deactivation in both task conditions (*u* = 2) or in at least one of the task conditions (*u* = 1). Multiple comparison corrections were performed using FDR and results were thresholded at *q* < 0.05. To identify differences between task conditions based on the dense data, we performed two-tailed paired *t*-tests with cluster size (*C* = 3.1) FWE multiple comparison correction. Permutation tests and multiple comparison correction were performed independently for left cortical surface, right cortical surface, and subcortical volume. To maintain whole-brain Type-I correction at .05, we set the threshold for the corrected *p*-values to *p* < 0.017. For group-level analysis on the parcellated data, we performed two-tailed one-sample *t*-tests with FDR correction to identify activation and deactivation during individual events. We also compared task conditions by performing two-tailed paired *t*-tests with FDR multiple comparison correction. The resulting images were thresholded at *q* < 0.05.

For the functional connectivity analysis, data were additionally processed to identify frames with excessive motion and associated signal artifacts and to remove nuisance signal. Specifically, we identified any frames in which motion exceeded 5 mm frame displacement or signal change was >1.6× the median of the normalized frame-to-frame root-mean-squared differences (RMSD) in voxel intensities. Identified frames were linearly interpolated before high-pass filtering (0.008 Hz) and excluded from further processing and analysis. We used a GLM approach to estimate the signal contributions from motion parameters, ventricular and white matter signal, global signal, and the first derivative of all the nuisance signals. To prevent systematic inflation of functional connectivity estimates due to task response (Cole et al., 2019), we also included task regressors using unassumed HRF task modeling across trial length (Experiment I: 14 frames, Experiment II: 32 frames) for each trial type separately. All functional connectivity analyses were performed on the residual signal after accounting for the listed nuisance and task regressors.

In the functional connectivity analyses, we focused on comparisons between pairs of task conditions across HCP parcels that were either identified as significantly activated during at least one of the task conditions or showed significant differences between these task conditions. We assigned parcel network membership based on the Cole-Anticevic Network Parcellation (Ji et al., 2019). We first constructed a delay-related time series for each identified parcel by concatenating those frames from each trial that best reflected the delay-related neural responses (Experiment I: frames 5–7 from trial onset, Experiment II: frames 10–15 from trial onset). This resulted in a *p* × *n* matrix, where *p* is the number of parcels and *n* is the number of delay-related frames across all trials within a condition. To avoid possible confounds, we excluded all frames from invalid trials, as well as trials where any of the frames met the criteria for motion scrubbing. We then computed Pearson's correlation coefficients between each pair of identified parcels across the delay-related time series, converted the correlation coefficients to Fisher-z values, and then averaged them to estimate functional connectivity within and between subnetworks for each participant separately. We performed group-level analysis using permutation analysis (10, 000 permutations) and FDR correction for multiple comparisons and presented results that exceeded the significance threshold of *q* < 0.05. This approach allowed us to focus our investigation specifically on delay-related functional integration across multiple ROIs and networks. The obtained estimates of functional connectivity reflect the frame-to-frame covariation of the BOLD signal during the information maintenance task phase after accounting for the mean task response, while also minimizing systematic effects due to motion and nuisance signals.

Group-level permutation analyses were performed in PALM (Winkler et al., 2014). Several steps of the analysis were performed using the Connectome Workbench (wb_command) tools. Visualization of the results was prepared in Connectome Workbench (wb_view), R (R Core Team, 2021), and BrainNet Viewer (Xia et al., 2013).

## 3. Results

### 3.1. Behavioral Performance

In the behavioral analysis, we had three goals. First, we wanted to check the extent to which the experimental manipulation of response predictability might have increased the difficulty of the task. Second, we wanted to check whether the previously observed response biases reflected features of spatial working memory representations or a motor response. Third, we wanted to assess possible differences in strategy use, specifically the use of categorical versus fine-grained representations between task conditions.

To test for possible differences in task difficulty due to manipulation of response predictability, we focused on error rates as a measure of behavioral performance under the *match* and *non-match* conditions. Specifically, we used logistic regression with the task condition (*match* vs. *non-match*) as a fixed and participants as a random factor to predict error rates. While we observed slightly higher error rates in the *non-match* compared to the *match* condition (see Figure 1B), a *Z*-test did not indicate the effect of the task condition to be statistically significant (β = 0.345, *Z* = 1.74, *p* = 0.083, *OR* = 0.708).

The *center* and *off-center* conditions allowed the assessment of more precise measures of response accuracy. Specifically, we calculated response accuracy as the standard deviation of the corrected angular and amplitude errors. In Experiment I, the permutation-based *t*-test revealed a significant difference between task conditions in the angular error, *t*_(22)_ = 2.80, *p* = 0.022, *d* = 0.503, reflecting slightly lower angular precision in the *off-center* condition (Figure 1B), but not for the amplitude error, *t*_(22)_ = 0.642, *p* = 0.529, *d* = 0.137. In contrast, the permutation-based *t*-test in Experiment II revealed a significant difference between task conditions in the amplitude error, *t*_(36)_ = 3.55, *p* = 0.002, *d* = 0.455, reflecting lower precision in the amplitude of the response in the *off-center* condition (Figure 1B), but not for angular error, *t*_(36)_ = 1.18, *p* = 0.256, *d* = 0.158.

Analysis of behavioral responses during *center* and *off-center* conditions revealed consistent systematic effects on response errors. Specifically, individual participants tended to either undershoot or overshoot their responses, which we estimated as a measure of systematic amplitude bias (Supplementary Figure 1). We also observed a robust systematic angular bias toward the nearest diagonal, which was most pronounced for target positions that were further from the nearest diagonal (Supplementary Figure 1). To assess the possibility that systematic biases reflect features of motor responses rather than working memory representations, we compared the magnitude of biases in the *center* and *off-center* conditions. In both experiments, amplitude bias did not differ significantly between task conditions (see Supplementary Figure 1B), Experiment I: *t*_(22)_ = 1.02, *p* = 0.318, *d* = 0.163; Experiment II: *t*_(36)_ = 0.328, *p* = 0.743, *d* = 0.031. We tested the differences in angular bias with a repeated-measures ANOVA with factors task condition (*center* vs. *off-center*) and target angle from the nearest diagonal (Experiment I: 0°, 10°, 20°, 30°, 40°; Experiment II: 7.5°, 22.5°, 37.5°). The results showed a significant effect of the target angle from the nearest diagonal on angular bias in both experiments, Experiment I: *F*_(4,88)_ = 23.8, *p* < 0.001, $\eta_{G}^{2}=0.261$; Experiment II: *F*_(2,72)_ = 93.3, *p* < 0.001, $\eta_{G}^{2}=0.340$, generally reflecting increasing angular bias toward diagonal with larger distance from the diagonal (see Supplementary Figure 1B). However, neither the main effect of task condition, Experiment I: *F*_(1,22)_ = 0.640, *p* = 0.263, $\eta_{G}^{2}=0.002$; Experiment II: *F*_(1,36)_ = 1.48, *p* = 0.068, $\eta_{G}^{2}=0.006$, nor the task condition × target angle interaction, Experiment I: *F*_(4,88)_ = 1.26, *p* = 0.288, $\eta_{G}^{2}=0.018$; Experiment II: *F*_(2,72)_ = 0.716, *p* = 0.488, $\eta_{G}^{2}=0.004$, showed evidence of significant differences, reflecting similar levels of angular bias independent of task condition.

### 3.2. Maintenance of Information in Spatial Working Memory Engages a Consistent Network of Brain Regions

**Figure 2:**
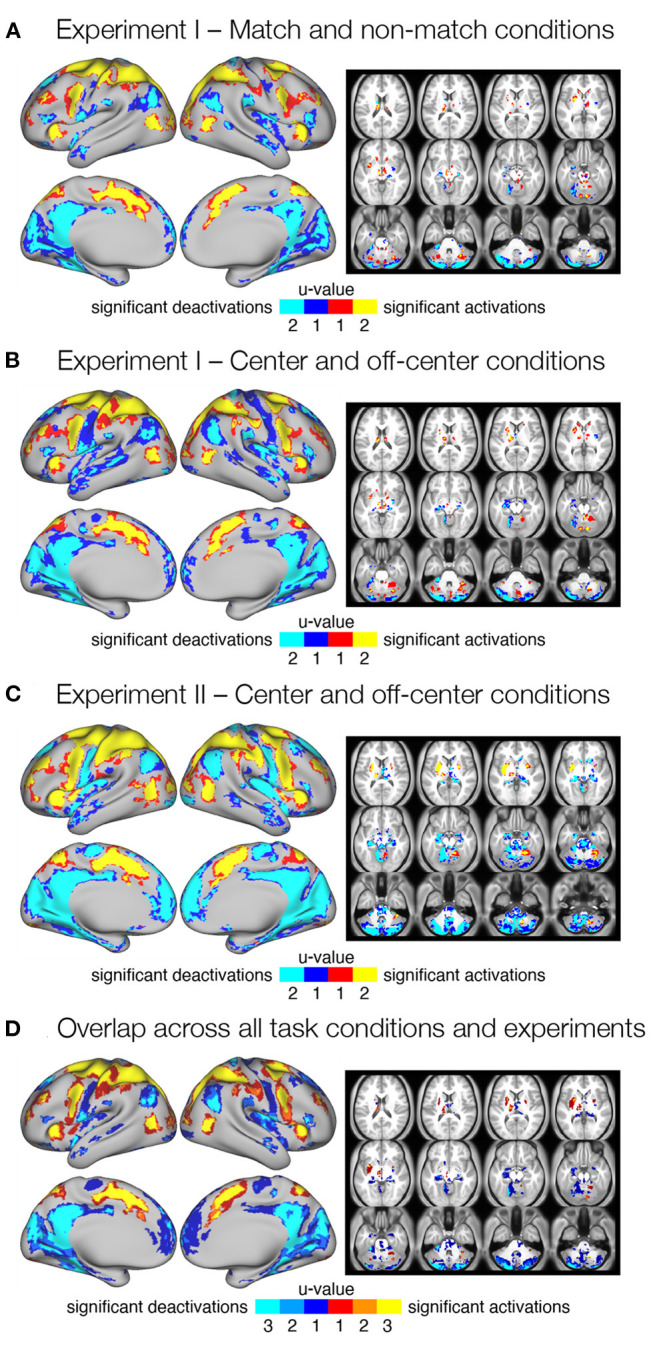
Significant delay-related responses. **(A–C)** Results of conjunction analysis showing significant delay-related activations (red-yellow) and deactivations (blue-cyan) in both (u = 2) or one (u = 1) of the task conditions for different task types and experiments, in particular **(A)** *match* and *non-match* conditions in Experiment I, **(B)** *center* and *off-center* conditions in Experiment I, and **(C)** *center* and *off-center* conditions in Experiment II. Shown are regions that were identified as significant at FDR corrected pooled *q* < 0.05. **(D)** Consistent delay-related activations and deactivations across all task conditions and experiments. The image was obtained by overlapping maps from individual conjunction analyses **(A–C)** that showed significant responses in both task conditions (*u* = 2). The resulting image shows delay-related activations (red-yellow) and deactivations (blue-cyan) present in one (*u* = 1), two (*u* = 2), or three (*u* = 3) maps from **(A–C)**.

In the initial step, we identified brain regions that were activated or deactivated during the delay of the spatial working memory task. We used conjunction analysis (Heller et al., 2007) to pool *p*-values from statistical maps across pairs of images with the same task type within the same experiment. Specifically, we identified grayordinates that showed a robust response in both task conditions (*u* = 2) as well as those that showed a significant response in at least one of the conditions (*u* = 1) at the FDR whole-brain corrected criterion for statistical significance *q* < 0.05. This resulted in three separate maps showing regions that robustly responded during the delay period in (i) *match* and/or *non-match* conditions in Experiment I (Figure 2A), (ii) *center* and/or *off-center* conditions in Experiment I (Figure 2B), and (iii) during *center* and/or *off-center* conditions in Experiment II (Figure 2C). Individual maps of Z-scores for each condition are shown in Supplementary Figure 2B. Comparison of regions that responded to one or both task conditions revealed differences mainly in spatial extent, likely due to a more stringent criterion.

To identify consistent activations and deactivations during the maintenance of spatial working memory information across task conditions and experiments, we combined all three maps of consistent responses across conditions (*u* = 2; Figures 2A–C) into a single overlap map (Figure 2D). The resulting image shows an overlap between none (*u* = 1), two (*u* = 2), or all three (*u* = 3) conjunction maps, and identifies a number of brain regions and systems that show robust delay-related responses in all conditions (*u* = 3). Specifically, increased activity was consistently observed in the somatosensory, motor, and premotor cortices. The superior and inferior parietal cortex, as well as several areas in the prefrontal and inferior frontal cortex, also showed consistent delay-related activation. Higher activity was also observed in the cingulate, insular, and opercular cortex, as well as in several higher visual areas. Subcortical activation was observed in the cerebellum (see Supplementary Table 1 for details). Decreased activity was consistently observed in posterior cingulate cortex, posterior opercular cortex, inferior and superior parietal cortex, medial temporal cortex, and insular cortex. Several sensory cortices, such as somatosensory, early and higher visual cortices, also showed decreased activity. Subcortical deactivation was most consistent in the cerebellum (Supplementary Table 2).

### 3.3. Task Conditions Are Associated With Differing Patterns of Delay Activity

Next, we examined whether the induced bias toward retrospective sensory coding in the *non-match* and *off-center* conditions resulted in observable differences in delay-related brain activity compared with the *match* and *center* conditions, which allowed either retrospective sensory or prospective motor coding.

**Figure 3:**
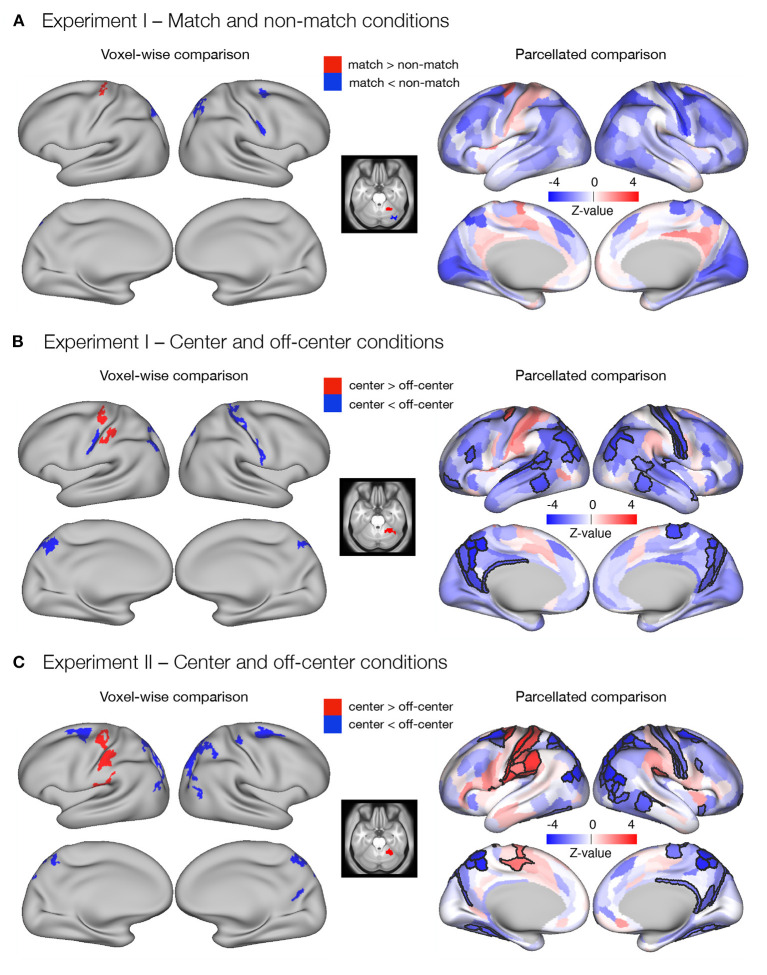
Differences in the delay activity between task conditions thought to promote either retrospective sensory or prospective motor coding, respectively. Specifically, dense and parcellated results are presented for contrasts between **(A)** *match* and *non-match* conditions and **(B)** *center* and *off-center* conditions in Experiment I, and **(C)** *center* and *off-center* conditions in Experiment II. Voxel-wise comparisons were obtained by comparing delay-period activity between task conditions using permutation *t*-tests on dense whole-brain data, corrected with cluster (*C* = 3.1) FWE, and thresholded at *p* < 0.05. For the parcellated comparisons, the dense whole-brain data were parcellated according to HCP-MMP1.0 parcellation (Glasser et al., 2016) before computing activity estimates. The significance of β estimates was computed using permutation *t*-tests and corrected for multiple comparisons with FDR. The resulting images of the parcellated data represent unthresholded maps of *Z*-values, while the black outlines mark the parcels that showed significant differences between task conditions, thresholded at *q* < 0.05.

The dense whole-brain comparison of *match* and *non-match* conditions in Experiment I (Figure 3A) revealed higher delay-related activity in the *match* condition in left premotor cortex, left supplementary motor area, and right cerebellum, and higher delay-related activity in the *non-match* condition in right premotor and motor cortex, right somatosensory cortex, bilateral superior parietal cortex, and right cerebellum (Supplementary Table 3). Investigation of parcellated data revealed no significant differences between *match* and *non-match* task conditions (Figure 3A).

The dense whole-brain comparison of *center* and *off-center* conditions in Experiment I (Figure 3B) showed higher delay-related activity in the *center* condition in left premotor and motor cortex, left somatosensory cortex, and right cerebellum, whereas right primary motor cortex, bilateral somatosensory cortex, bilateral superior parietal cortex, left inferior parietal cortex, and bilateral posterior cingulate cortex showed significantly higher delay-related activity in the *off-center* condition (Supplementary Table 4). Analysis of parcellated data revealed several additional significant differences (Figure 3B). In particular, in the *center* condition, higher delay-related activity was observed in the left frontal opercular cortex. In the *off-center* condition, higher activity was observed bilaterally in early and association auditory cortices, lateral temporal cortex, and temporo-parieto-occipital junction. Left-lateralized differences were observed in a number of prefrontal regions and in superior temporal visual area, whereas right-lateralized differences were found in the posterior opercular cortex and inferior parietal cortex (Supplementary Table 6).

In Experiment II, dense whole-brain comparison of delay-related response between the *center* and *off-center* conditions (Figure 3C) revealed higher activity in left premotor and motor cortex, left somatosensory cortex, left posterior opercular cortex, left inferior parietal cortex, and right cerebellum during the *center* condition. During the *off-center* condition, significantly higher activity was observed bilaterally in premotor cortex, inferior parietal cortex, superior parietal cortex, posterior cingulate cortex, and dorsal stream of visual cortex. Right-lateralized differences were observed in primary motor cortex, somatosensory cortex, and temporo-parieto-occipital junction (Supplementary Table 5). Analysis of parcellated data revealed many additional brain areas that showed significant differences (Figure 3C). Activity was higher during *center* in the left supplementary motor area, left cingulate motor area, bilateral frontal opercular cortex, and right posterior opercular cortex (Supplementary Table 7). During *off-center*, activity was higher in a number of right-lateralized regions in prefrontal cortex (PFC), sensory-visual, and association areas (Supplementary Table 9). Left-lateralized differences were observed in the temporo-parieto-occipital junction, whereas bilateral changes occurred in the medial temporal cortex and the ventral stream visual cortex (Supplementary Table 8).

Direct comparison of difference maps across task types and experiments indicated moderate to large similarities (*r* = 0.43–0.52) in the identified differences of brain responses (see Supplementary Figure 4A for details).

### 3.4. Task Conditions Are Associated With Differences in Temporal Progression of Delay-Related Responses

**Figure 4:**
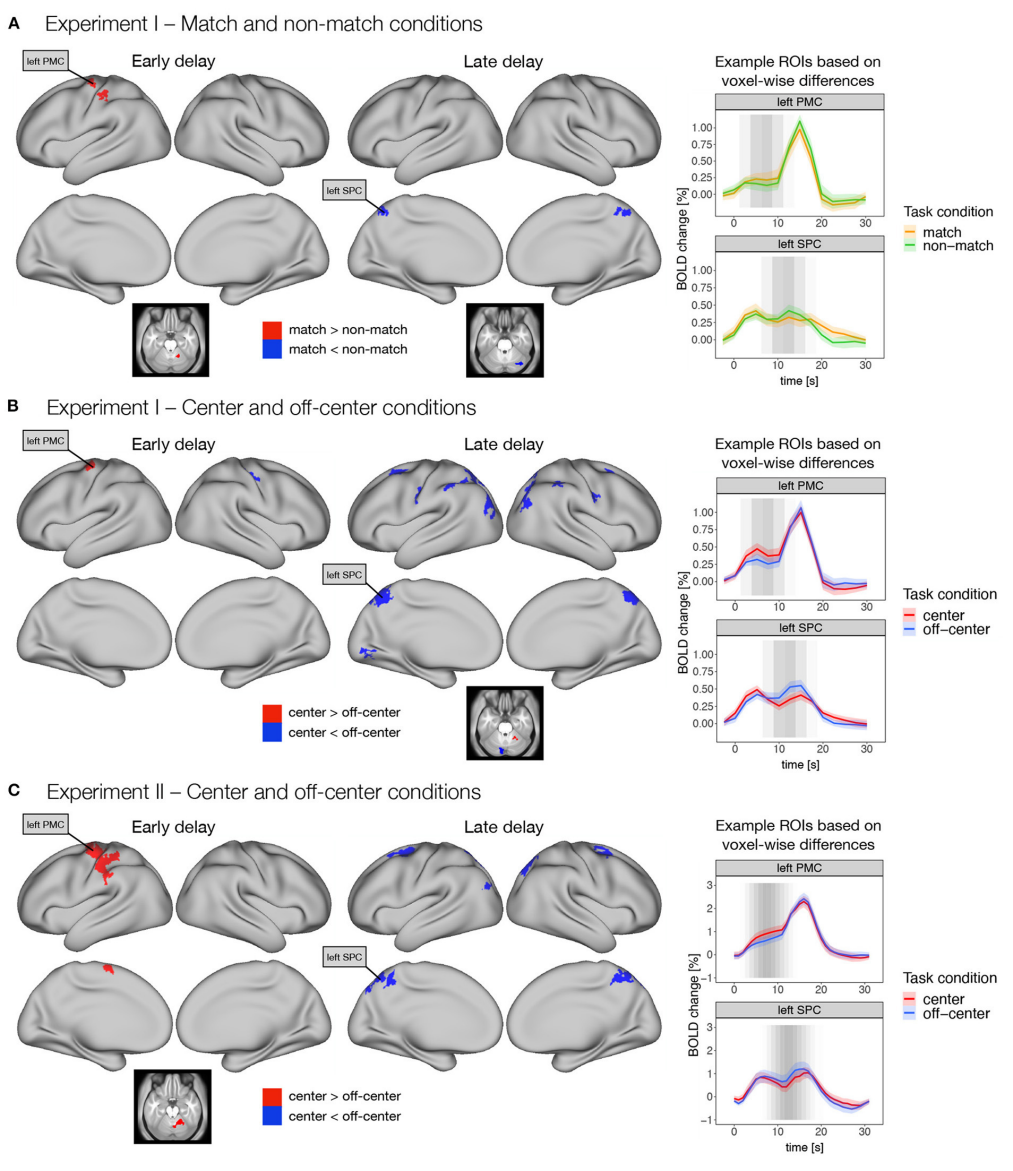
Differences in early and late phases of the delay activity between task conditions thought to promote either retrospective sensory or prospective motor coding, respectively. Specifically, dense whole-brain results are presented for contrasts between **(A)** *match* and *non-match* conditions, and **(B)** *center* and *off-center* conditions in Experiment I, and **(C)** *center* and *off-center* conditions in Experiment II. Early delay was defined as activity in the first half of the delay period, and late delay was defined as activity in the second half of the delay period. Differences between task conditions were calculated using permutation *t*-tests on dense whole-brain data, corrected with cluster (*C* = 3.1) FWE, and thresholded at *p* < 0.05. Based on the thresholded images, we identified two example ROIs (i.e., left premotor cortex—PMC and left superior parietal cortex—SPC) that exhibited significant differences across different task conditions and experiments. We then extracted the average activity within each ROI throughout the entire trial for each task condition. The zero point represents the onset of target presentation. The gray shading in the plots marks the expected contribution of the early and late delay periods to the reconstructed trial response based on the hemodynamic response function.

Different coding and representation strategies may be associated with different temporal progression during a working memory trial. To observe temporal progression of the differences in delay-related response, we modeled the delay activity as two separate events. We modeled the first half of the delay period as early delay and the second half of the delay period as late delay (see Supplementary Figure 2A). We then again contrasted task conditions with different assumed coding mechanisms for both phases of the delay period (Figure 4). In particular, we were interested in the comparisons of (i) *match* and *non-match* conditions and (ii) *center* and *off-center* conditions in Experiment I and (iii) *center* and *off-center* conditions in Experiment II.

Comparison of the *match* and *non-match* conditions in Experiment I (Figure 4A) showed higher activity in the *match* condition in left premotor and motor cortex, left somatosensory cortex, and right cerebellum during the early delay. In contrast, higher activity in the *non-match* condition was observed in bilateral superior parietal cortex, right posterior cingulate cortex, and right cerebellum during late delay (Supplementary Table 3).

Comparison of the *center* and *off-center* conditions in Experiment I (Figure 4B) showed higher activity in the *center* condition in left premotor cortex during the early delay and in right cerebellum during the late delay. In contrast, activity in the *off-center* condition was higher in the right somatosensory cortex during the early delay, whereas in the late delay, activity in the *off-center* condition was higher in a number of brain areas, namely, bilaterally in premotor cortex, somatosensory cortex, DLPFC, inferior parietal cortex, superior parietal cortex, posterior cingulate cortex, and early and higher visual areas. Higher activity during the late delay for *off-center* was also observed in right primary motor cortex and left cerebellum (Supplementary Table 4).

In Experiment II, higher activity in the *center* condition was again observed only during the early delay in areas within the left premotor cortex, left primary motor cortex, left supplementary motor area, left somatosensory cortex, left inferior parietal cortex, and right cerebellum (Figure 4C). In contrast, in the *off-center* condition, higher activity was observed in bilateral premotor cortex, bilateral posterior cingulate cortex, bilateral superior parietal cortex, left inferior parietal cortex, and right dorsal stream of visual cortex only during the late delay (Figure 4C, Supplementary Table 5).

To further investigate these differences, we extracted an average time course for the previously identified regions throughout the trial. The extracted time courses showed consistent temporal changes during the different task conditions. Specifically, higher activity in the *match* and *center* conditions was mostly observed earlier during the delay period, whereas higher activity in the *non-match* and *off-center* conditions was observed later in the delay period (Figure 4).

Direct comparison of whole-brain difference maps across task types and experiments indicated small to moderate similarities (*r* = 0.14–0.28) in the early and moderate to large similarities (*r* = 0.30–0.48) in the late delay phases (see Supplementary Figures 4B,C for details).

### 3.5. Task Manipulation Is Associated With Changes in Functional Connectivity

Finally, we expected that different strategies of encoding and maintenance of spatial information in working memory would require integration of activity and exchange of information between different brain regions and systems. To test this hypothesis, we investigated possible differences in task-related functional connectivity between areas found to be engaged in spatial working memory. We selected parcels for functional connectivity analysis based on activation analysis of the parcellated data. Specifically, we included in the analysis all parcels that were either significantly activated during any of the task conditions (Supplementary Figure 3) or showed significant differences between task conditions (Figure 3, right panel). We then estimated functional connectivity between the selected parcels across the delay-related time series and examined functional connectivity within and between brain subnetworks based on the network membership of the parcels.

**Figure 5:**
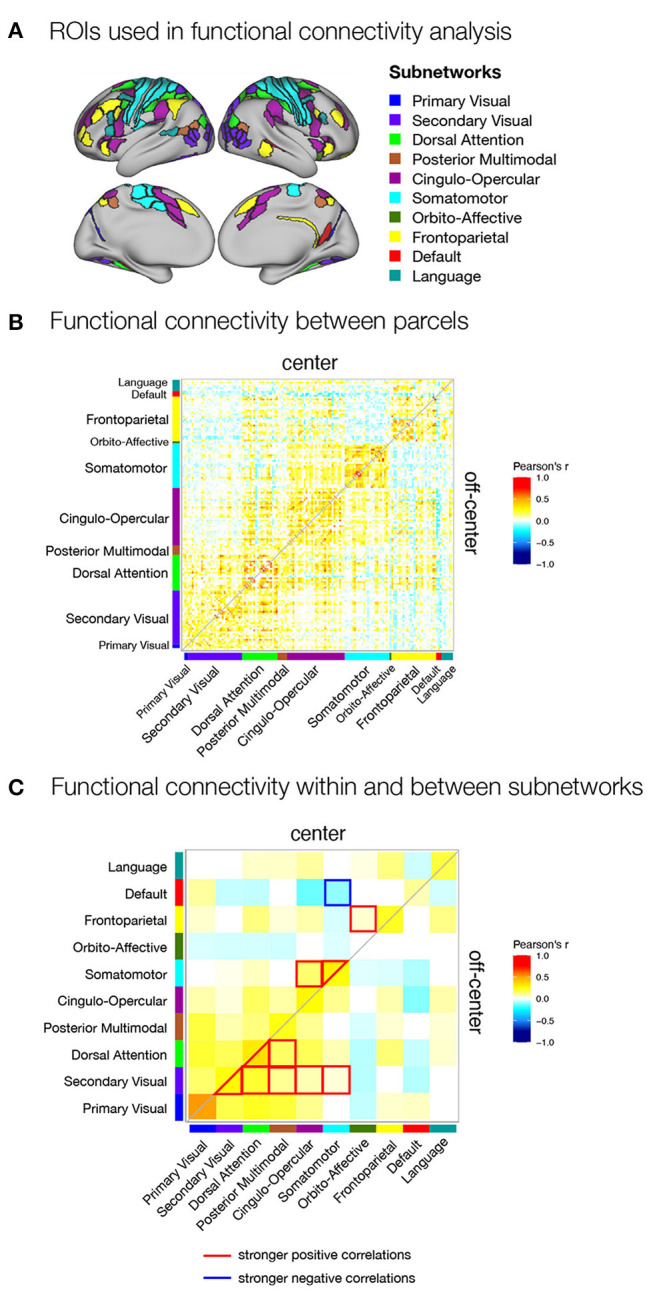
Functional connectivity within the spatial working memory network during the delay period of *center* and *off-center* conditions in Experiment II. **(A)** ROIs selected for functional connectivity analysis. ROIs were identified based on activation analysis as any region that was active during the delay period of any of the task conditions or showed differences in delay activity between the same task conditions. ROIs were then assigned to subnetworks as described by Ji et al. (2019). Functional connectivity during the delay **(B)** between individual parcels for each task condition and **(C)** between subnetworks for each task condition and differences between task conditions. As a measure of functional connectivity, we calculated Pearson's correlation coefficients between the delay activity of individual ROIs or specific subnetworks. Statistically significant correlations were identified using permutation analysis, using FDR to control for multiple comparisons and thresholding results at *q* < 0.05. The resulting correlation matrices are not symmetric: they show Pearson's correlation coefficients for the *center* condition in the upper triangle and for the *off-center* condition in the lower triangle.

We focused in particular on comparisons between the *match* and *non-match* (Supplementary Figure 5A) and *center* and *off-center* (Supplementary Figure 5B) conditions in Experiment I and *center* and *off-center* conditions in Experiment II (Figure 5, Supplementary Figure 6). The results revealed very similar patterns of functional connectivity across task conditions. Statistical tests for differences in functional connectivity between task conditions revealed a number of significant differences only in Experiment II. Comparison of functional connectivity between individual parcels (Supplementary Figure 6) showed generally higher positive correlations between somatomotor, premotor, and secondary visual areas and stronger negative correlations between frontal and posterior sensory areas during the *center* condition compared with *off-center*. In comparison, the *off-center* condition was characterized by higher positive correlations within areas in parietal cortex and between frontal and parietal cortices. Apart from isolated differences within frontal and parietal cortex, we did not observe a general pattern of stronger negative correlations in the *off-center* condition.

We obtained a clearer pattern of differences when we compared the average functional connectivity between and within subnetworks in *center* and *off-center* conditions in Experiment II (Figure 5C). In the *center* condition, we primarily observed stronger connectivity involving somatomotor network, which showed stronger within-network connectivity, stronger connectivity with the cingulo-opercular network, and stronger anti-correlations with the default network. We also observed significantly increased connectivity between fronto-parietal and orbito-affective networks. In contrast, the *off-center* condition was characterized by stronger connectivity between and within the secondary visual and dorsal attentional networks and their connectivity with the posterior multimodal network. The secondary visual network also showed an increase in functional connectivity with cingulo-opercular and somatomotor networks.

## 4. Discussion

Performing a spatial working memory task engages a number of cognitive processes and representations that enable the perception, encoding, maintenance, retrieval, and use of spatial information. While a number of these processes are required regardless of the specific demands of the task, in this study we explored the possibility that the specific form of spatial representation used may depend on the information available and the demands of the current task. To test this possibility, we designed a spatial working memory task that manipulated available information required to successfully perform the task. Specifically, improving on previous studies by Curtis et al. (2004) and Srimal and Curtis (2008), we manipulated the predictability of the motor response required to perform the task while keeping the required level of precision and the type of response constant across conditions. We expected participants to use prospective motor coding to a greater extent when the motor response was known at the time of target presentation. In contrast, if the motor response could not be predicted, participants would have to rely on retrospective sensory coding.

To test our hypotheses, we first identified the core working memory network, i.e., the set of regions that are robustly engaged irrespective of task manipulation and related differences in the use of working memory strategies and representations. Next, we focused on investigation of observable differences between key task conditions. We explored differences in the overall engagement of brain regions, differences in the dynamics of activation across different phases of the trial, and differences in the integration of information within and between those parcels of the brain networks that were engaged by the working memory task. In the following sections, we review the corresponding results.

### 4.1. Engagement of the Core Spatial Working Memory Network

Spatial working memory engaged a widespread network of brain areas, which we observed as elevated activity that persisted throughout the delay period. The observation of sustained activity during the delay is consistent with much of the literature on spatial working memory and suggests its role in spatial working memory performance. In our study, we observed robust activity in areas extending across the frontal and parietal cortices to posterior sensory areas and several subcortical structures, such as cerebellum. These areas were consistently activated in two separate experiments reported in this article under task conditions designed to promote the use of different encoding and maintenance strategies. The observed results suggest the existence of a core spatial working memory network that supports spatial encoding and maintenance and is engaged independently of specific task requirements.

The areas activated during delay observed in our study are consistent with the results of other studies that have used similar spatial working memory tasks (Zarahn et al., 1999; Brown et al., 2004; Curtis et al., 2004; Srimal and Curtis, 2008). Indeed, many of the areas reported in our study are also found in other types of working memory tasks using different features and stimulus modalities. For example, a coordinate-based meta-analysis of 189 experiments by Rottschy et al. (2012) showed consistent activation during a variety of working memory tasks in both hemispheres in the posterior medial frontal cortex, anterior insula, IPS, lateral PFC, and inferior frontal gyrus (IFG). This observation suggests that these areas may be responsible for more general support processes related to working memory. Nevertheless, the distributed nature of spatial working memory indicates distinct functional roles of the areas involved in storing spatial representations (Rissman and Wagner, 2012; Eriksson et al., 2015; Christophel et al., 2017). As spatial information passes through different levels of processing, from incoming sensory processing to action planning, the areas responsible for particular levels of processing could use different encoding and maintenance strategies (Christophel et al., 2017).

It is important to note the similarities of the identified network to previous studies despite specific task differences. Though our task was based on paradigms first used in electrophysiological studies in monkeys (Funahashi et al., 1989, 1993; Takeda and Funahashi, 2002, 2004) and later in neuroimaging studies in humans (Curtis et al., 2004; Srimal and Curtis, 2008), most of them have used saccades to provide task responses. The advantage of using saccades is that they are well-studied and activation of the FEF, thought to support preparation and execution of saccades (Robinson and Fuchs, 1969; Bruce and Goldberg, 1985; Thier and Andersen, 1998), was observed during delay in spatial working memory tasks (Bruce and Goldberg, 1985; Brown et al., 2004; Curtis et al., 2004). In contrast, participants in our study used hand movements to move an object on a screen with a joystick. We believe that the use of hand movements resulted in activation of premotor areas that are superior to the FEF and may be related to hand movement control. However, we also observed delay-related activation of the FEF itself. These results suggest that multiple encoding and maintenance strategies may have been employed simultaneously in our task. We address this possibility in the *Significance and limitations* section.

### 4.2. Strategy Specific Activations of Brain Regions

The spatial working memory task in our study was designed to contrast two conditions, one that allowed encoding and maintenance of either retrospective sensory information or a prospective motor plan (*match* or *center* condition), and another that prevented the use of prospective motor codes and forced participants to rely solely on retrospective sensory information (*non-match* or *off-center* condition). We found that this manipulation of strategy use was reflected in brain activity. In general, somatomotor, premotor, and motor areas were more active when participants were able to use motor representations, whereas somatomotor and parietal areas were more active when they were required to rely primarily on sensory representations. These results were replicated in different task types and across both experiments. Similar results were also obtained by Curtis et al. (2004), who showed higher activity in FEF, SEF, and MFG during the task that enabled prospective motor coding and in IPS during the task that biased toward retrospective sensory coding.

The task conditions that allowed the use of prospective motor coding showed robust activity in frontal areas related to the preparation of motor goals and their execution. Although these task conditions allowed the use of retrospective sensory coding, our results suggest that participants used motor related strategies either exclusively or simultaneously with retrospective sensory coding. In the case of our study, the required motor responses were hand movements, which was likely related to the higher activity observed in superior portions of the premotor cortex. Studies that investigated different coding strategies, have predominantly observed the role of FEF in preparing prospective saccade targets during spatial working memory (Bruce and Goldberg, 1985; Curtis et al., 2004) and neural responses in the FEF have been found to be topographically organized in response to visual stimuli or saccade direction (Bruce and Goldberg, 1985; Jerde et al., 2012). Other studies have shown that FEF is activated even in the absence of saccades (Curtis et al., 2004; Srimal and Curtis, 2008), suggesting that the strategy of encoding spatial information in the form of saccade plans could be used even in the absence of actual saccades. In our study, we did not observe differences in FEF activity between task conditions. This could be due to a number of possibilities. First, because no condition required saccade responses, participants might not have used motor planning of saccades in any of the conditions. Second, prospective motor planning of saccades could have been used in all conditions as an additional supportive strategy for task performance. And third, as suggested by Srimal and Curtis (2008), FEF activity could reflect the maintenance of the general spatial saliency map that was used in all task conditions rather than saccade motor plans.

In contrast, task conditions that required retrospective sensory coding were most strongly associated with higher activity in parietal areas. Parietal cortex has been consistently associated with representation of visual space and spatial attention. Single-cell recordings in animals have shown robust persistent delay-period activity in parietal neurons spatially selective for remembered stimulus location in spatial working memory tasks (e.g., Chafee and Goldman-Rakic, 1998; Gottlieb and Goldberg, 1999; Zhang and Barash, 2004). To date, up to seven distinct topographic areas encoding visual space have been discovered in the posterior parietal cortex (PPC), six along the IPS and the seventh in the superior parietal lobule (Kastner et al., 2007; Silver and Kastner, 2009; Medendorp et al., 2011; Jerde et al., 2012). Moreover, parietal cortex has been consistently activated during covert spatial attention tasks (Corbetta et al., 1998; Bisley and Goldberg, 2003; Ikkai and Curtis, 2008; Szczepanski et al., 2010; Caspari et al., 2015; Bogadhi et al., 2018). Consistent with previous studies, our results support the proposition that sensory spatial representations are encoded in parietal areas and maintained by covert, spatially directed attention.

While a number of areas in frontal and parietal cortex showed differences in delay-related activity between task conditions, it should be noted that their activity was not specific to any single condition. Rather, the regions were engaged in both conditions, albeit to different extents. We can assume that their contribution to specific encoding and maintenance strategies changes according to task demands. This observation is supported by experiments with single-cell recordings in animals, where the same population of neurons can encode both retrospective sensory information and a prospective motor code, even within the same trial (Zhang and Barash, 2000, 2004; Takeda and Funahashi, 2002, 2004; Medendorp et al., 2005). The differences between task conditions might also reflect different demands on some common support processes necessary to maintain spatial information in working memory. For example, the fronto-parietal cortex has been implicated in a variety of executive functions in addition to working memory, such as attention, cognitive control, and motor control (Theeuwes et al., 2009; Ikkai and Curtis, 2011; Jonikaitis and Moore, 2019), all of which have been proposed as possible mechanisms for working memory maintenance. Based on our results, it is difficult to determine whether the differences in activity are due to the use of condition specific representations or to differences in the extent of involvement in more general support processes.

### 4.3. Strategy Dependent Temporal Dynamics During Delay Period

Differences in the strategies used to support task performance were reflected not only in overall delay-related activity, but also in their temporal dynamics within a trial. During task conditions that enabled prospective motor coding, higher activity was observed primarily at the beginning of the delay period, whereas retrospective sensory coding was associated with higher activity later in the delay period. These results are consistent with the hypothesis that prospective motor coding involves the recoding of perceptual information into a prospective motor plan early in the trial with stimulus-related activity decreasing in the later part of the trial. In contrast, reliance on retrospective sensory information requires consistent maintenance of sensory information and reactivation before responding must occur.

The dynamics observed in our task contrast with results from single-cell recordings in monkeys, where Takeda and Funahashi (2004) observed that DLPFC cells encoded the direction of the cue early in the trial, which transformed into coding the direction of the saccade only late during the 3 s delay. Our results are though consistent with human studies in which repetitive transcranial magnetic stimulation (rTMS) of the DLPFC and PPC was performed at different phases of delay during an oculomotor delayed-response task (Muri et al., 1996; Brandt et al., 1998). In both studies, rTMS over PPC was found to significantly reduce task precision only when applied during the initial, sensory, or encoding phase of the trial, 50 ms (Brandt et al., 1998) and 260 ms (Muri et al., 1996) after cue presentation, and not during the later, memory-related phase of the trial, 360 ms or later after cue onset. The opposite was true for rTMS over the DLPFC. Given the proposed role of the PPC in stimulus processing and the DLPFC in memory maintenance, these results suggest rapid recoding and reduced reliance on sensory information in the delay phase in a task that allows early recoding of sensory information into a motor plan.

Similar temporal dynamics were observed in the original (Curtis et al., 2004) study. The early delay period was characterized by increased activation of areas associated with motor planning of saccades in the *match* condition, whereas the *non-match* condition resulted in higher activation of regions associated with sensory information in the late delay period.

The results of this and previous studies suggest that, when possible, initial sensory information is rapidly transformed into representations that are easier to robustly maintain across the delay period. This is evident in the initial increase in activation in conditions that support the conversion, which is specific to regions associated with the response representation. Once transcoding is complete, it does not appear to require an additional increase in maintenance-related activation during the later phases of the delay. In contrast, if sensory information must be relied upon, its active maintenance persists throughout the delay period, or additional processing associated with reactivation of the information might be required before the response is executed.

### 4.4. Strategy Related Integration Within the Spatial Working Memory Network

Early neuroimaging studies, including studies of spatial working memory, focused primarily on the functional localization of cognitive processes. Studies in the past two decades have, however, shown that most cognitive tasks require successful engagement and integration of information across a network of brain regions. A series of experiments has illustrated the importance of structural connectivity and functional interactions between regions commonly associated with spatial working memory (e.g., Blatt et al., 1990; Stanton et al., 1995; Matelli et al., 1998; Gazzaley et al., 2004; Curtis et al., 2005; Miller and D'Esposito, 2012; Braunlich et al., 2015). It is therefore reasonable to assume that different cognitive strategies in task performance are also reflected in different functional connectivity between regions engaged during spatial working memory task, possibly even without observable differences in mean activation levels.

Our investigation of delay-related functional connectivity indicated a consistent pattern of functional connectivity across conditions. Significant differences between the patterns of functional connectivity within and between the key brain networks in the two conditions became apparent only in Experiment II, possibly due to more robust estimates of functional connectivity made possible by higher temporal resolution (1 vs. 2.5 s TR) and higher statistical power due to a larger number of participants (37 vs. 23) and a generally larger number of trials.

The results of the direct comparison of functional connectivity between the two conditions indicated a more prominent role of the somatomotor network when participants were biased toward the use of prospective motor coding of the response. Specifically, in the *center* condition, we observed stronger functional connectivity within the somatomotor network, increased functional connectivity of the somatomotor network with the cingulo-opercular network, and stronger anti-correlations with the default network. The *off-center* condition, which required reliance solely on retrospective sensory information, was associated with stronger integration between and within the secondary visual and dorsal attentional networks, whose functional connectivity with the posterior multimodal, cingulo-opercular, and somatomotor networks was increased.

The observed pattern of differences in functional connectivity is consistent with the proposition that predictability of a motor response allows participants to prepare and maintain a motor plan, which requires integration of information within sensorimotor regions, maintenance of sensorimotor network engagement by the cingulo-opercular cognitive control network (Dosenbach et al., 2006), and reduced interference by the default network. Similarly, the need to rely primarily on visual sensory information led to greater integration of information within the visual system and the attentional network. The stronger integration with the dorsal attentional network is consistent with the long-standing observation that visual attention and spatial working memory share resources (Oh and Kim, 2004) and the hypothesis that revisiting spatial locations by covert attention may serve as a mechanism to refresh locations maintained in spatial working memory (for a review see Repovs and Baddeley, 2006). The increase in functional connectivity between secondary visual and cingulo-opercular networks underpins both the proposed role of the cingulo-opercular network in maintenance of relevant task sets and the difference between task sets in the two task conditions.

Our functional connectivity findings are also consistent with the observations by Curtis et al. (2005), who focused on comparing the coherence of an FEF seed with the rest of the brain between task conditions that encouraged either retrospective sensory or prospective motor coding. Their results showed increased coherence of FEF with SEF and dorsal anterior cingulate cortex during the prospective motor coding condition, and in contrast, greater coherence of FEF with DLPFC, superior frontal sulcus, and PPC during retrospective sensory coding. Taken together, these results suggest that differences in task conditions lead to the use of different strategies for encoding and maintenance of spatial information, which are reflected not only in the activity of different brain regions, but also in the patterns of integration of information within and between brain functional connectivity networks.

### 4.5. Significance and Limitations

Our results contribute to a growing literature showing that multiple strategies and representations can be used to encode and maintain information in a working memory task. Furthermore, they show that the specific strategies used can be directed or biased by task conditions, allowing the identification of associated neural mechanisms, brain systems, and networks. They also suggest that the available strategies are not mutually exclusive and that their use may reflect or depend on individual differences.

Whereas, previous studies of coding spatial working memory information have focused primarily on memory guided saccades, our study focused on manual responses using a joystick. This offered a number of advantages. First, it allowed us to decouple the sensory and response reference points, which enabled independent manipulation of response predictability, task difficulty, and the memory processes used in a way that is not possible in a task requiring memory-guided saccades. Second, the results of the study are applicable to understanding a broader range of spatial working memory studies, since manual responses are easier to perform and therefore more common compared to studies of memory-guided saccades that require eye-tracking, especially in the context of fMRI and in studies with specific populations such as children and some patient populations. Third, our results show that a simple manipulation of the response starting position significantly alters the task performance strategies used. In the case of the *off-center* condition, the task effectively eliminates the use of prospective motor coding, reducing the variability in cognitive strategies and associated cognitive and neural processes across task conditions and individuals.

When interpreting the results and drawing conclusions from this study, it is important to keep in mind that the study design only directly manipulated the ability to prepare and maintain the hand response. Participants were still able and free to use other supporting strategies and representations to maintain spatial information. One of these strategies is the use of coarse categorical information. Behavioral results indicated a clear presence of bias toward the diagonal, which was present in both *center* and *off-center* conditions. Because there were no differences in the magnitude of bias between task conditions, we can conclude that the task design did not affect the use of the categorical representation and therefore was not reflected in the observed differences in activation patterns.

Another strategy that could be used by participants is covert planning of eye movements. Participants were instructed not to move their eyes during the presentation of the target and the delay phases of the trial. However, they could use covert saccade planning as a supportive memorization strategy by guiding the response disk to the memorized saccade target location in the response phase. The use of oculomotor planning in tasks that did not explicitly require eye movements has been identified in previous studies (e.g., Ball et al., 2013; Pearson et al., 2014). Again, this strategy could be used in both the *center* and *off-center* conditions, and thus should not contribute to the observed differences between the two task conditions. However, we cannot rule out the possibility that some of the FEF activation is due to such memorization strategy.

An important limitation of the current study is the lack of continuous real-time control of eye movements, which would allow strict adherence to gaze on the fixation point during the presentation of the cue and delay. In the absence of such control, participants could potentially use unpredictable strategies, such as fixating gaze on the position of the cue and moving the response disk to the position of gaze. Such uncontrolled eye movements could interfere with the purely mnemonic activity and prevent reliable interpretation of our results. This would be particularly problematic if eye movements were used more frequently in only one of the two contrasting conditions. In Experiment II, we were able to address these concerns by checking the presence and frequency of inappropriate eye movements offline using the simultaneously recorded EOG signal. We did detect the occasional occurrence of eye movements during the presentation of the target and the delay periods of the task, but we found no significant differences in their occurrence between the *center* and *off-center* conditions. Based on these results, we believe that the observed differences in brain activity between task conditions are due to factors other than eye movements.

Relatedly, we have not explicitly controlled hand movements during the delay. This allows for the possibility that participants made small preparatory hand movements during the delay, which could contribute to the observed activity in somatomotor brain areas. While this possibility can not be excluded, participants were instructed to move their hands during the response only and no participant reported the use of such strategy in the in-depth debriefing on the strategies used after the scanning sessions.

An additional limitation to consider is the difference between the *center* and *off-center* stimulus presentations in the response phase. Whereas, in the *center* condition the gray response disk appeared in the center of the screen, at the position of the participant's gaze, in the *off-center* condition the response disk appeared at a random position that was not central to the participant's gaze. In the latter case, the response disk could present a distractor contributing to increased delay-related activity, as observed in previous studies on the effects of distractors in visual working memory (e.g., Dolcos et al., 2007). However, in evaluating this possibility, it is important to consider, first, that the increases in activity in the *off-center* condition were observed primarily in the late delay period, whereas in previous studies (Dolcos et al., 2007), they were present throughout the delay period. Second, the increases were also observed in the *non-match* condition compared with *match*, which did not differ in the response phase stimulus presentation. Finally, similar increases were also observed in the Curtis et al. (2004) study, which also did not contain distracting stimuli in the response phase. Future studies should investigate this possibility more directly.

Finally, it is important to note differences in the trial progression between the two experiments reported in the paper. Specifically, experiments differed in whether a fixation cue was provided at the beginning of a trial (Experiment I) or not (Experiment II). The presence of the fixation cue enables participants to fixate their gaze before target presentation and efficiently encode a brief stimulus, potentially improving their performance and reducing attentional engagement during the ITI. The fixation cue does, however, present a sensory event in itself, which elicits separate sensory and attentional processes that could affect estimation of target encoding processes. Importantly, the results indicate that the presence or absence of the fixation cue did not affect delay-related estimates of interest.

## 5. Conclusion

In this study, we aimed to investigate whether slight changes in the spatial working memory task lead to the use of different strategies for encoding and maintenance of spatial information and whether we can observe their specific neural correlates. The results suggest that although the same core spatial working memory network is activated regardless of task conditions, the relative activation of individual brain regions, the dynamics of their response across trial duration, and the integration of brain activity within and between brain networks support the hypothesis that participants can encode and maintain spatial information by prospective representation of a motor response or a retrospective representation of sensory information. In the case of prospective motor coding, the motor code is generated early in the delay period and its maintenance is supported by the integration of information with and within the sensorimotor brain network. In the case of retrospective sensory coding, the maintenance of information is supported by integration with and within secondary visual and dorsal attentional networks and reactivated late in the delay period before a response is required. These results support the proposition that spatial working memory is not realized by a single representational system, but that spatial information in working memory can be encoded and maintained by multiple complementary representations, cognitive processes, and associated brain systems.

## Data Availability Statement

The behavioral data, EOG data and fMRI ROI activity and functional connectivity data and analysis scripts are available in OSF repository: https://osf.io/497ej. Whole-brain statistical maps are available in BALSA repository: https://balsa.wustl.edu/study/x2rpz.

## Ethics Statement

The studies involving human participants were reviewed and approved by the Ethics Committee, Faculty of Arts, University of Ljubljana, Ljubljana, Slovenia, and the National Medical Ethics Committee, Ministry of Health, Republic of Slovenia, Ljubljana, Slovenia. The patients/participants provided their written informed consent to participate in this study.

## Author Contributions

NP, MS, AS, and GR: conceptualization. NP, MS, and GR: methodology. NP, AK, AM, and GR: software. NP: formal analysis, writing—original draft, visualization, and project administration. NP, MS, AS, AK, and AM: investigation. NP, AS, AK, AM, and GR: writing—review and editing. GR: supervision and funding acquisition. All authors contributed to the article and approved the submitted version.

## Funding

This work was supported by the Slovenian Research Agency (Young Researcher program and the research grants J7-5553, J3-9264, P3-0338, P5-0110).

## Conflict of Interest

GR consults for and holds equity in Neumora Therapeutics and Manifest Technologies. The remaining authors declare that the research was conducted in the absence of any commercial or financial relationships that could be construed as a potential conflict of interest.

## Publisher's Note

All claims expressed in this article are solely those of the authors and do not necessarily represent those of their affiliated organizations, or those of the publisher, the editors and the reviewers. Any product that may be evaluated in this article, or claim that may be made by its manufacturer, is not guaranteed or endorsed by the publisher.

## References

[B1] Awh E. Jonides J. (2001). Overlapping mechanisms of attention and spatial working memory. Trends Cogn. Sci. 5, 119–126. 10.1016/S1364-6613(00)01593-X 11239812

[B2] Ball K. Pearson D. G. Smith D. T. (2013). Oculomotor involvement in spatial working memory is task-specific. Cognition 129, 439–446. 10.1016/j.cognition.2013.08.006 24001480

[B3] Bisley J. W. Goldberg M. E. (2003). Neuronal activity in the lateral intraparietal area and spatial attention. Science 299, 81–86. 10.1126/science.1077395 12511644

[B4] Blatt G. J. Andersen R. A. Stoner G. R. (1990). Visual receptive field organization and cortico-cortical connections of the lateral intraparietal area (area LIP) in the macaque. J. Comp. Neurol. 299, 421–445. 10.1002/cne.902990404 2243159

[B5] Bogadhi A. R. Bollimunta A. Leopold D. A. Krauzlis R. J. (2018). Brain regions modulated during covert visual attention in the macaque. Sci. Rep. 8:15237. 10.1038/s41598-018-33567-9 30323289 PMC6189039

[B6] Brandt S. A. Ploner C. J. Meyer B.-U. Leistner S. Villringer A. (1998). Effects of repetitive transcranial magnetic stimulation over dorsolateral prefrontal and posterior parietal cortex on memory-guided saccades. Exp. Brain Res. 118, 197–204. 10.1007/s002210050272 9547088

[B7] Braunlich K. Gomez-Lavin J. Seger C. A. (2015). Frontoparietal networks involved in categorization and item working memory. NeuroImage 107, 146–162. 10.1016/j.neuroimage.2014.11.051 25482265 PMC4306569

[B8] Brown M. DeSouza J. Goltz H. C. Ford K. Menon R. S. Goodale M. A.. (2004). Comparison of memory- and visually guided saccades using event-related fMRI. J. Neurophysiol. 91, 873–889. 10.1152/jn.00382.2003 14523078

[B9] Bruce C. J. Goldberg M. E. (1985). Primate frontal eye fields. I. Single neurons discharging before saccades. J. Neurophysiol. 53, 603–635. 10.1152/jn.1985.53.3.603 3981231

[B10] Caspari N. Janssens T. Mantini D. Vandenberghe R. Vanduffel W. (2015). Covert shifts of spatial attention in the macaque monkey. J. Neurosci. 35, 7695–7714. 10.1523/JNEUROSCI.4383-14.2015 25995460 PMC4438122

[B11] Chafee M. V. Goldman-Rakic P. S. (1998). Matching patterns of activity in primate prefrontal area 8a and parietal area 7ip neurons during a spatial working memorytask. J. Neurophysiol. 79, 2919–2940. 10.1152/jn.1998.79.6.2919 9636098

[B12] Christophel T. B. Klink P. C. Spitzer B. Roelfsema P. R. Haynes J.-D. (2017). The distributed nature of working memory. Trends Cogn. Sci. 21, 111–124. 10.1016/j.tics.2016.12.007 28063661

[B13] Cole M. W. Ito T. Schultz D. Mill R. Chen R. Cocuzza C. (2019). Task activations produce spurious but systematic inflation of task functional connectivity estimates. NeuroImage 189, 1–18. 10.1016/j.neuroimage.2018.12.054 30597260 PMC6422749

[B14] Corbetta M. Akbudak E. Conturo T. E. Snyder A. Z. Ollinger J. M. Drury H. A.. (1998). A common network of functional areas for attention and eye movements. Neuron 21, 761–773. 10.1016/S0896-6273(00)80593-0 9808463

[B15] Curtis C. (2006). Prefrontal and parietal contributions to spatial working memory. Neuroscience 139, 173–180. 10.1016/j.neuroscience.2005.04.070 16326021

[B16] Curtis C. E. Rao V. Y. D'Esposito M. (2004). Maintenance of spatial and motor codes during oculomotor delayed response tasks. J. Neurosci. 24, 3944–3952. 10.1523/JNEUROSCI.5640-03.2004 15102910 PMC6729424

[B17] Curtis C. E. Sun F. T. Miller L. M. D'Esposito M. (2005). Coherence between fMRI time-series distinguishes two spatial working memory networks. NeuroImage 26, 177–183. 10.1016/j.neuroimage.2005.01.040 15862217

[B18] Dolcos F. Miller B. Kragel P. Jha A. McCarthy G. (2007). Regional brain differences in the effect of distraction during the delay interval of a working memory task. Brain Res. 1152, 171–181. 10.1016/j.brainres.2007.03.059 17459348 PMC3514456

[B19] Dosenbach N. U. F. Visscher K. M. Palmer E. D. Miezin F. M. Wenger K. K. Kang H. C.. (2006). A core system for the implementation of task sets. Neuron 50, 799–812. 10.1016/j.neuron.2006.04.031 16731517 PMC3621133

[B20] Eriksson J. Vogel E. K. Lansner A. Bergström F. Nyberg L. (2015). Neurocognitive architecture of working memory. Neuron 88, 33–46. 10.1016/j.neuron.2015.09.020 26447571 PMC4605545

[B21] Friston K. J. Jezzard P. Turner R. (1994). Analysis of functional MRI time-series. Hum. Brain Mapp. 1, 153–171. 10.1002/hbm.460010207

[B22] Funahashi S. (2015). Functions of delay-period activity in the prefrontal cortex and mnemonic scotomas revisited. Front. Syst. Neurosci. 9:2. 10.3389/fnsys.2015.00002 25698942 PMC4318271

[B23] Funahashi S. Bruce C. J. Goldman-Rakic P. S. (1989). Mnemonic coding of visual space in the monkey's dorsolateral prefrontal cortex. J. Neurophysiol. 61, 331–349. 10.1152/jn.1989.61.2.331 2918358

[B24] Funahashi S. Chafee M. V. Goldman-Rakic P. S. (1993). Prefrontal neuronal activity in rhesus monkeys performing a delayed anti-saccade task. Nature 365, 753–756. 10.1038/365753a0 8413653

[B25] Fuster J. M. (1973). Unit activity in prefrontal cortex during delayed-response performance: neuronal correlates of transient memory. J. Neurophysiol. 36, 61–78. 10.1152/jn.1973.36.1.61 4196203

[B26] Gazzaley A. Rissman J. D'Esposito M. (2004). Functional connectivity during working memory maintenance. Cogn. Affect. Behav. Neurosci. 4, 580–599. 10.3758/CABN.4.4.580 15849899

[B27] Glasser M. F. Coalson T. S. Robinson E. C. Hacker C. D. Harwell J. Yacoub E.. (2016). A multi-modal parcellation of human cerebral cortex. Nature 536, 171–178. 10.1038/nature18933 27437579 PMC4990127

[B28] Glasser M. F. Sotiropoulos S. N. Wilson J. A. Coalson T. S. Fischl B. Andersson J. L.. (2013). The minimal preprocessing pipelines for the Human Connectome Project. NeuroImage 80, 105–124. 10.1016/j.neuroimage.2013.04.127 23668970 PMC3720813

[B29] Gottlieb J. Goldberg M. E. (1999). Activity of neurons in the lateral intraparietal area of the monkey during an antisaccade task. Nat. Neurosci. 2, 906–912. 10.1038/13209 10491612

[B30] Haun D. B. Allen G. L. Wedell D. H. (2005). Bias in spatial memory: a categorical endorsement. Acta Psychol. 118, 149–170. 10.1016/j.actpsy.2004.10.011 15627414

[B31] Heller R. Golland Y. Malach R. Benjamini Y. (2007). Conjunction group analysis: an alternative to mixed/random effect analysis. NeuroImage 37, 1178–1185. 10.1016/j.neuroimage.2007.05.051 17689266

[B32] Huttenlocher J. Hedges L. V. Corrigan B. Crawford L. (2004). Spatial categories and the estimation of location. Cognition 93, 75–97. 10.1016/j.cognition.2003.10.006 15147930

[B33] Huttenlocher J. Hedges L. V. Duncan S. (1991). Categories and particulars: prototype effects in estimating spatial location. Psychol. Rev. 98, 352–376. 10.1037/0033-295X.98.3.352 1891523

[B34] Ikkai A. Curtis C. E. (2008). Cortical activity time locked to the shift and maintenance of spatial attention. Cereb. Cortex 18, 1384–1394. 10.1093/cercor/bhm171 17921456 PMC2629659

[B35] Ikkai A. Curtis C. E. (2011). Common neural mechanisms supporting spatial working memory, attention and motor intention. Neuropsychologia 49, 1428–1434. 10.1016/j.neuropsychologia.2010.12.020 21182852 PMC3081523

[B36] Jerde T. A. Merriam E. P. Riggall A. C. Hedges J. H. Curtis C. E. (2012). Prioritized maps of space in human frontoparietal cortex. J. Neurosci. 32, 17382–17390. 10.1523/JNEUROSCI.3810-12.2012 23197729 PMC3544526

[B37] Ji J. L. Demšar J. Fonteneau C. Tamayo Z. Pan L. Kraljič A.. (in preparation). QuNex-A scalable platform for integrative multi-modal neuroimaging data processing analysis.

[B38] Ji J. L. Spronk M. Kulkarni K. Repovš G. Anticevic A. Cole M. W. (2019). Mapping the human brain's cortical-subcortical functional network organization. NeuroImage 185, 35–57. 10.1016/j.neuroimage.2018.10.006 30291974 PMC6289683

[B39] Jonikaitis D. Moore T. (2019). The interdependence of attention, working memory and gaze control: behavior and neural circuitry. Curr. Opin. Psychol. 29, 126–134. 10.1016/j.copsyc.2019.01.012 30825836

[B40] Kastner S. DeSimone K. Konen C. S. Szczepanski S. M. Weiner K. S. Schneider K. A. (2007). Topographic maps in human frontal cortex revealed in memory-guided saccade and spatial working-memory tasks. J. Neurophysiol. 97, 3494–3507. 10.1152/jn.00010.2007 17360822

[B41] Matelli M. Govoni P. Galletti C. Kutz D. F. Luppino G. (1998). Superior area 6 afferents from the superior parietal lobule in the macaque monkey. J. Compar. Neurol. 402, 327–352. 10.1002/(SICI)1096-9861(19981221)402:3<327::AID-CNE4>3.0.CO;2-Z 9853903

[B42] Medendorp W. P. Buchholz V. N. Van Der Werf J. Leoné F. T. M. (2011). Parietofrontal circuits in goal-oriented behaviour. Eur. J. Neurosci. 33, 2017–2027. 10.1111/j.1460-9568.2011.07701.x 21645097

[B43] Medendorp W. P. Goltz H. C. Vilis T. (2005). Remapping the remembered target location for anti-saccades in human posterior parietal cortex. J. Neurophysiol. 94, 734–740. 10.1152/jn.01331.2004 15788514

[B44] Miller B. T. D'Esposito M. (2012). Spatial and temporal dynamics of cortical networks engaged in memory encoding and retrieval. Front. Hum. Neurosci. 6:109. 10.3389/fnhum.2012.00109 22557959 PMC3340945

[B45] Muri R. M. Vermersch A. I. Rivaud S. Gaymard B. Pierrot-Deseilligny C. (1996). Effects of single-pulse transcranial magnetic stimulation over the prefrontal and posterior parietal cortices during memory-guided saccades in humans. J. Neurophysiol. 76, 2102–2106. 10.1152/jn.1996.76.3.2102 8890321

[B46] Niki H. Watanabe M. (1976). Prefrontal unit activity and delayed response: relation to cue location versus direction of response. Brain Res. 105, 79–88. 10.1016/0006-8993(76)90924-0 1252960

[B47] Oh S.-H. Kim M.-S. (2004). The role of spatial working memory in visual search efficiency. Psychon. Bull. Rev. 11, 275–281. 10.3758/BF03196570 15260193

[B48] Pearson D. G. Ball K. Smith D. T. (2014). Oculomotor preparation as a rehearsal mechanism in spatial working memory. Cognition 132, 416–428. 10.1016/j.cognition.2014.05.006 24908341

[B49] Peirce J. W. (2007). PsychoPy-psychophysics software in python. J. Neurosci. Methods 162, 8–13. 10.1016/j.jneumeth.2006.11.017 17254636 PMC2018741

[B50] Postle B. (2006). Working memory as an emergent property of the mind and brain. Neuroscience 139, 23–38. 10.1016/j.neuroscience.2005.06.005 16324795 PMC1428794

[B51] R Core Team (2021). R: A Language and Environment for Statistical Computing. Vienna: R Foundation for Statistical Computing.

[B52] Rainer G. Asaad W. F. Miller E. K. (1998). Memory fields of neurons in the primate prefrontal cortex. Proc. Natl. Acad. Sci. U.S.A. 95, 15008–15013. 10.1073/pnas.95.25.15008 9844006 PMC24566

[B53] Repovs G. Baddeley A. (2006). The multi-component model of working memory: explorations in experimental cognitive psychology. Neuroscience 139, 5–21. 10.1016/j.neuroscience.2005.12.061 16517088

[B54] Rissman J. Wagner A. D. (2012). Distributed representations in memory: insights from functional brain imaging. Annu. Rev. Psychol. 63, 101–128. 10.1146/annurev-psych-120710-100344 21943171 PMC4533899

[B55] Robinson D. A. Fuchs A. F. (1969). Eye movements evoked by stimulation of frontal eye fields. J. Neurophysiol. 32, 637–648. 10.1152/jn.1969.32.5.637 4980022

[B56] Rottschy C. Langner R. Dogan I. Reetz K. Laird A. Schulz J.. (2012). Modelling neural correlates of working memory: a coordinate-based meta-analysis. NeuroImage 60, 830–846. 10.1016/j.neuroimage.2011.11.050 22178808 PMC3288533

[B57] Silver M. A. Kastner S. (2009). Topographic maps in human frontal and parietal cortex. Trends Cogn. Sci. 13, 488–495. 10.1016/j.tics.2009.08.005 19758835 PMC2767426

[B58] Srimal R. Curtis C. E. (2008). Persistent neural activity during the maintenance of spatial position in working memory. NeuroImage 39, 455–468. 10.1016/j.neuroimage.2007.08.040 17920934 PMC2219966

[B59] Stanton G. B. Bruce C. J. Goldberg M. E. (1995). Topography of projections to posterior cortical areas from the macaque frontal eye fields. J. Comp. Neurol. 353, 291–305. 10.1002/cne.903530210 7745137

[B60] Starc M. Anticevic A. Repovš G. (2017). Fine-grained versus categorical: pupil size differentiates between strategies for spatial working memory performance. Psychophysiology 54, 724–735. 10.1111/psyp.12828 28127779

[B61] Szczepanski S. M. Konen C. S. Kastner S. (2010). Mechanisms of spatial attention control in frontal and parietal cortex. J. Neurosci. 30, 148–160. 10.1523/JNEUROSCI.3862-09.2010 20053897 PMC2809378

[B62] Takeda K. Funahashi S. (2002). Prefrontal task-related activity representing visual cue location or saccade direction in spatial working memory tasks. J. Neurophysiol. 87, 567–588. 10.1152/jn.00249.2001 11784772

[B63] Takeda K. Funahashi S. (2004). Population vector analysis of primate prefrontal activity during spatial working memory. Cereb. Cortex 14, 1328–1339. 10.1093/cercor/bhh093 15166104

[B64] Theeuwes J. Belopolsky A. Olivers C. N. (2009). Interactions between working memory, attention and eye movements. Acta Psychol. 132, 106–114. 10.1016/j.actpsy.2009.01.005 19233340

[B65] Thier P. Andersen R. A. (1998). Electrical microstimulation distinguishes distinct saccade-related areas in the posterior parietal cortex. J. Neurophysiol. 80, 1713–1735. 10.1152/jn.1998.80.4.1713 9772234

[B66] Winkler A. M. Ridgway G. R. Webster M. A. Smith S. M. Nichols T. E. (2014). Permutation inference for the general linear model. NeuroImage 92, 381–397. 10.1016/j.neuroimage.2014.01.060 24530839 PMC4010955

[B67] Xia M. Wang J. He Y. (2013). BrainNet viewer: a network visualization tool for human brain connectomics. PLoS ONE 8:e68910. 10.1371/journal.pone.0068910 23861951 PMC3701683

[B68] Zarahn E. Aguirre G. K. D'Esposito M. (1999). Temporal isolation of the neural correlates of spatial mnemonic processing with fMRI. Cogn. Brain Res. 7, 255–268. 10.1016/S0926-6410(98)00029-9 9838152

[B69] Zhang M. Barash S. (2000). Neuronal switching of sensorimotor transformations for antisaccades. Nature 408, 971–975. 10.1038/35050097 11140683

[B70] Zhang M. Barash S. (2004). Persistent LIP activity in memory antisaccades: working memory for a sensorimotor transformation. J. Neurophysiol. 91, 1424–1441. 10.1152/jn.00504.2003 14523076

