# Supplementary material for: Neural Evidence for Different Types of Position Coding Strategies in Spatial Working Memory
Source: Front Hum Neurosci. 2022 Apr 20;16:821545. doi: 10.3389/fnhum.2022.821545 (PMC9067305; doi:10.3389/fnhum.2022.821545)
Supplement: Supplementary file 1 [file Data_Sheet_1.pdf]

Supplementary Material

1 SUPPLEMENTARY METHODS

1.1 EEG acquisition and EOG analysis

In both experiments, participants were instructed to look at the center of the screen during the presentation of the target and the delay period, while they were free to move their eyes during the response and the inter-trial interval. However, participants' gaze was not monitored and controlled in real time. This could be problematic because eye movements and gazing angles have been shown to have strong effects on activity in brain regions commonly associated with working memory processes, and without eye movement control, it would be more difficult to distinguish between pure working memory activation and eye movement related activity (Funahashi, 2015). Nonetheless, simultaneous fMRI-EEG recording in Experiment II enabled the study of eye movements using electrooculography (EOG) signal extracted from the EEG data.

EEG data were recorded simultaneously with the fMRI BOLD images using a Brain Products MR compatible EEG system with 128 electrodes (BrainAmp MR, Brain Products GmbH, Gilching, Germany). The impedance of the electrodes was kept below 15 kΩ. Data were recorded at a sampling rate of 5000 Hz. During recording, high-pass filtering with a time constant of 10 s and low-pass filtering with a cutoff frequency of 250 Hz were performed. The recorded signals for all electrodes were referenced to the vertex electrode (FCz).

Preprocessing and analysis of EEG data was performed in Matlab (R2020a, Natick, Massachusetts). First, imaging artifacts were removed with the average artifact subtraction algorithm using 25 averaging windows (Niazy et al., 2005; Allen et al., 2000). For movement-related MR-induced artifact removal, we used the carbon wire loop system as described by van der Meer et al. (2016). We further preprocessed the EEG signal to remove power line noise using the CleanLine method (Mullen, 2012), and a bandpass filter between 0.01 Hz and 100 Hz was applied (Hamming windowed-sinc FIR filter). The signal from each channel was re-referenced to the average signal and downsampled to 500 Hz. The horizontal EOG signal was calculated from the difference between the left (F9) and right (F10) temporal electrodes. For the vertical EOG signal, the electrodes above the left (Fp1) and right (Fp2) eyes were averaged. To detect horizontal and vertical eye movements, we used the free-viewing saccade detection method described by Jia and Tyler (2019).

First, we excluded any participants (total 6 participants) or individual sessions that had incomplete EEG recordings or poor data quality. We also excluded all epochs with poor data quality and all trials that were not used to estimate the BOLD signal. The detection of saccades from the EOG signal showed that participants generally did not move their eyes on more than 7.81% (median, IQR $= 15.8\%$) of trials during the *center* condition and 11.1% (median, IQR $= 16.7\%$) of trials during the *off-center* condition. Based on these results, we assumed that participants generally followed instructions (including those for whom we had no eye movement information), and we considered all participants for further analyses.

Because the analysis of the behavioral and fMRI data focused primarily on differences between task conditions, we wanted to rule out the possibility that a difference between conditions was either an artefact of, or masked by, the difference in saccade occurrence. We used logistic regression to test for differences in saccade frequency and found no evidence of a systematic effect of task condition ($\beta = 0.258$, $Z = 1.87$, $p = .061$, $OR = 1.29$).

2 SUPPLEMENTARY FIGURES

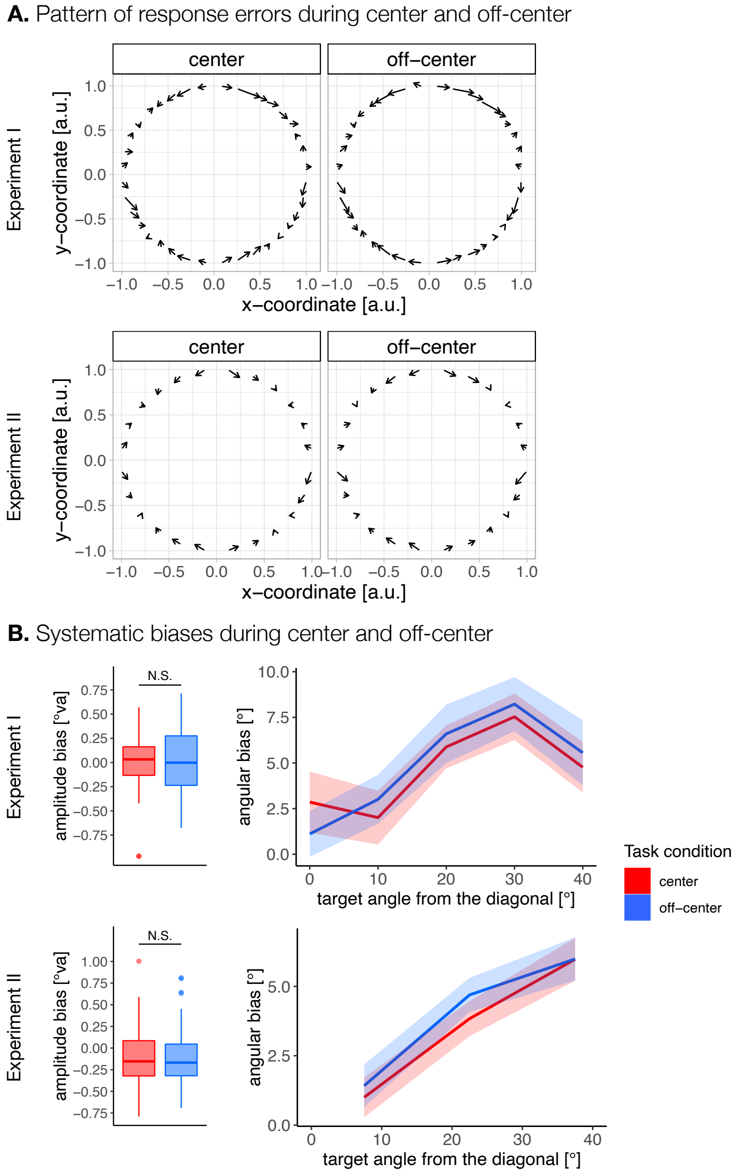

Figure S1. Patterns of response errors and average systematic biases during *center* and *off-center* conditions. A. The averaged response errors at different target locations for the *center* and *off-center* conditions in both experiments. Investigation of the response patterns revealed systematic biases in the amplitude and angular errors. The results showed that individual subjects tended to either overshoot or undershoot their responses on average, which was reflected in the amplitude error. In addition, subjects tended to shift their responses closer to the nearest diagonals, which was reflected in larger angular errors at target angles further from the nearest diagonal. **B.** The average amplitude bias for each task condition and the average angular bias at different target angles from the nearest diagonal for each task condition. N.S. – not significant.

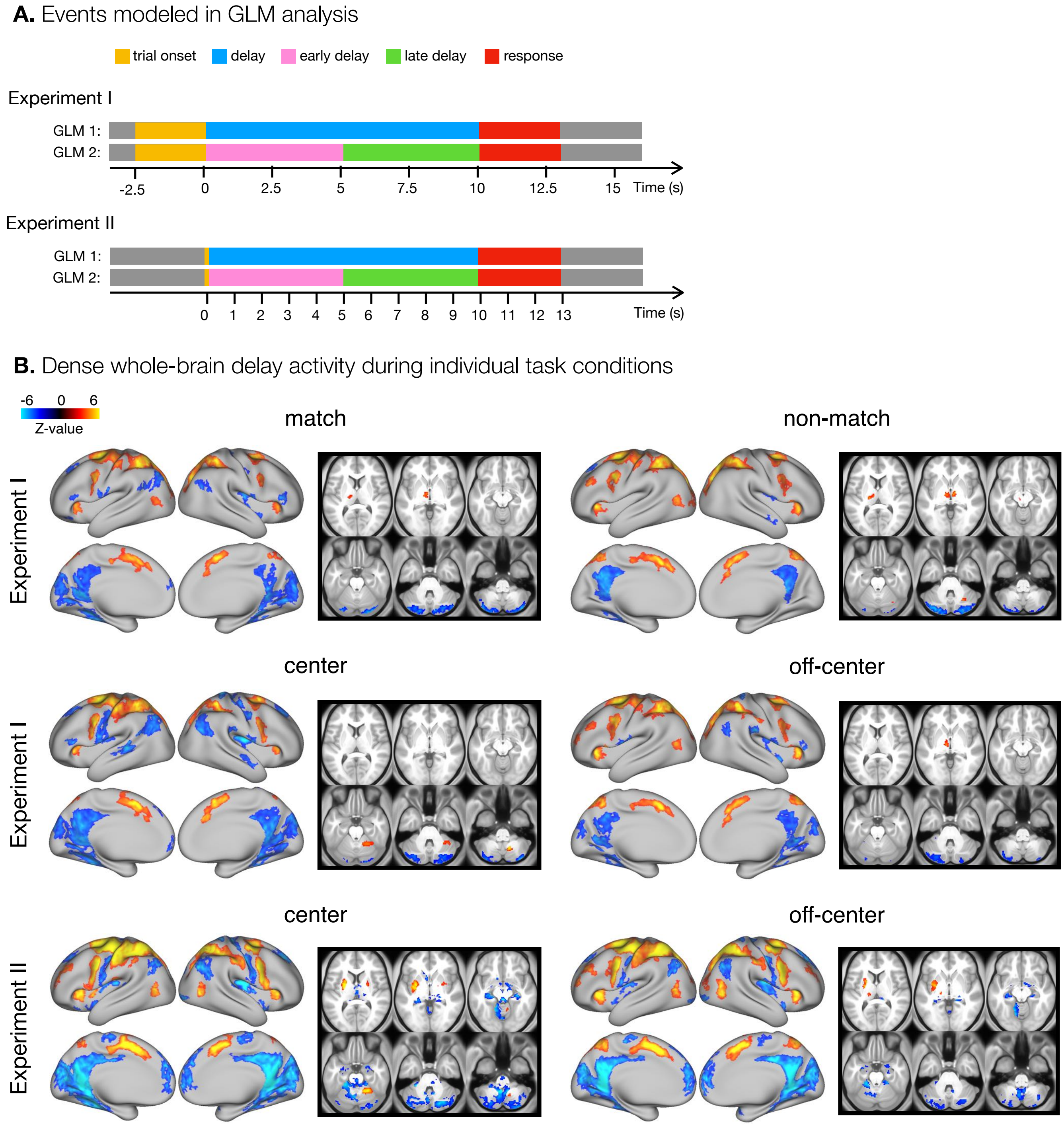

Figure S2. Events modeled in GLM analysis and average delay activity for individual task conditions based on the dense whole-brain analysis. A. The timing and duration of events modeled in the GLM analysis of the fMRI data. First, we performed the GLM analysis with events trial onset, delay, and response for both experiments. Next, we decomposed the delay period into separate regressors for early and late delay. **B.** The average delay activity during individual task conditions for both experiments. Images were obtained by a one-sample t-test of the GLM regressor during the delay period, corrected with cluster $(C = 3.1)$ FWE, and thresholded at $p < 0.05$.

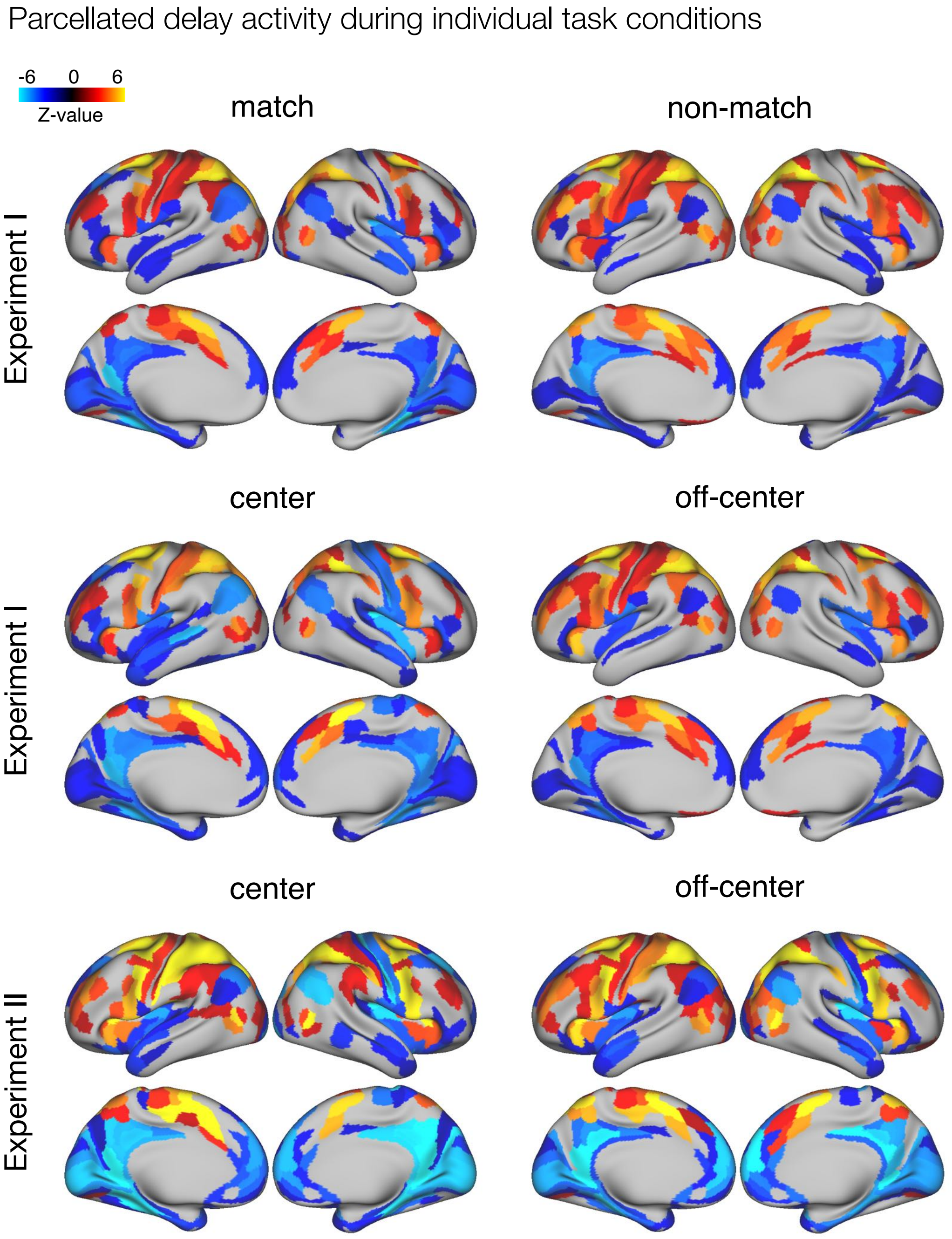

Figure S3. Average delay activity during individual task conditions based on analysis of parcellated data. The images show the average delay activity during individual task conditions within specific HCP parcels for both experiments. Results were obtained by a one-sample t-test of the GLM regressor during the delay period, corrected with FDR and thresholded at $q < 0.05$.

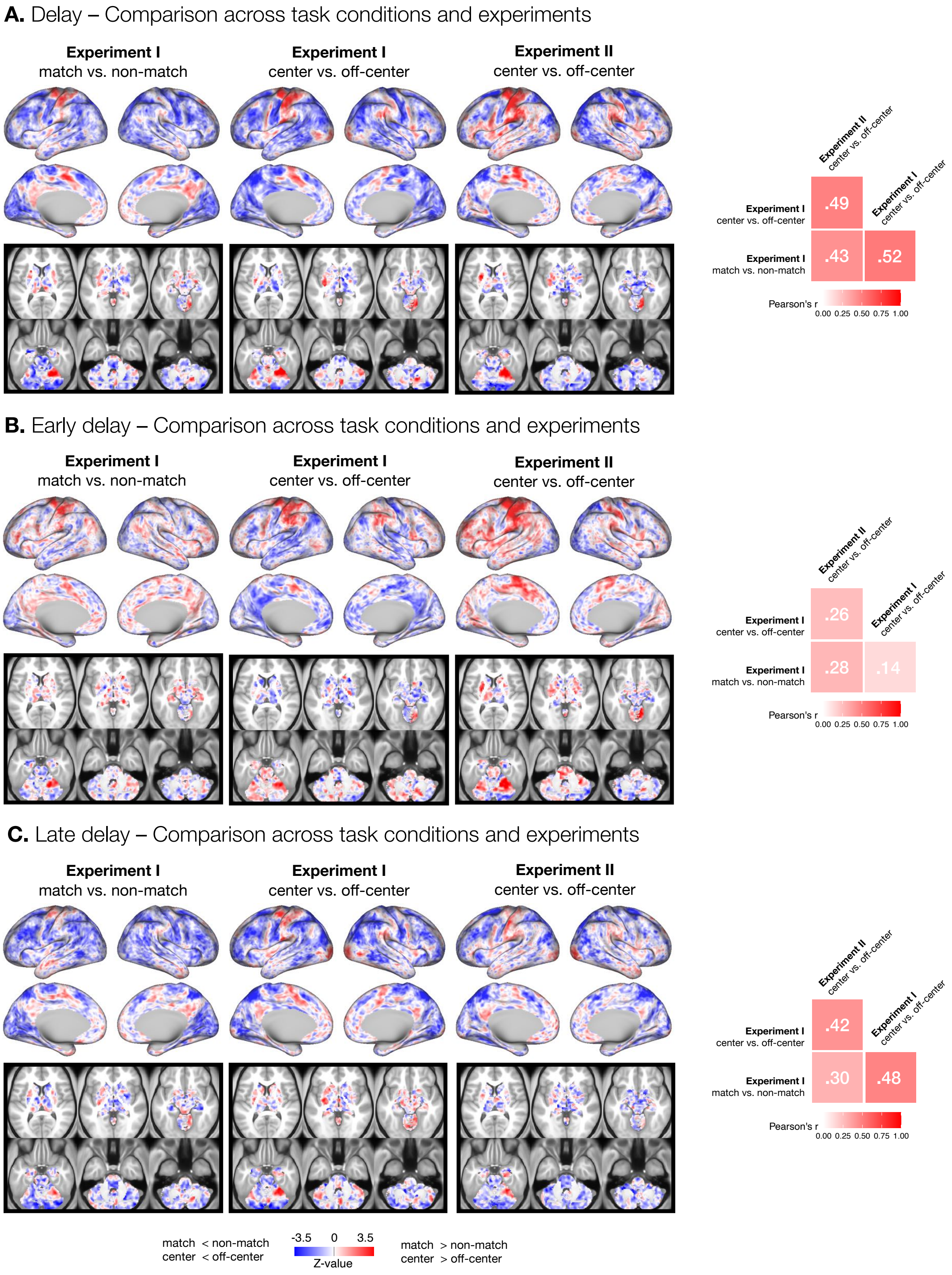

Figure S4. Consistency of delay-related differences across different task conditions and experiments. Presented are unthresholded Z-maps of either **A.** delay, **B.** early delay or **C.** late delay activity differences between specific pairs of task conditions, namely *match* and *non-match* conditions, and *center* and *off-center* conditions in Experiment I, and *center* and *off-center* conditions in Experiment II. The right panel shows related Pearson's correlation coefficients between different pairs of unthresholded Z-maps.

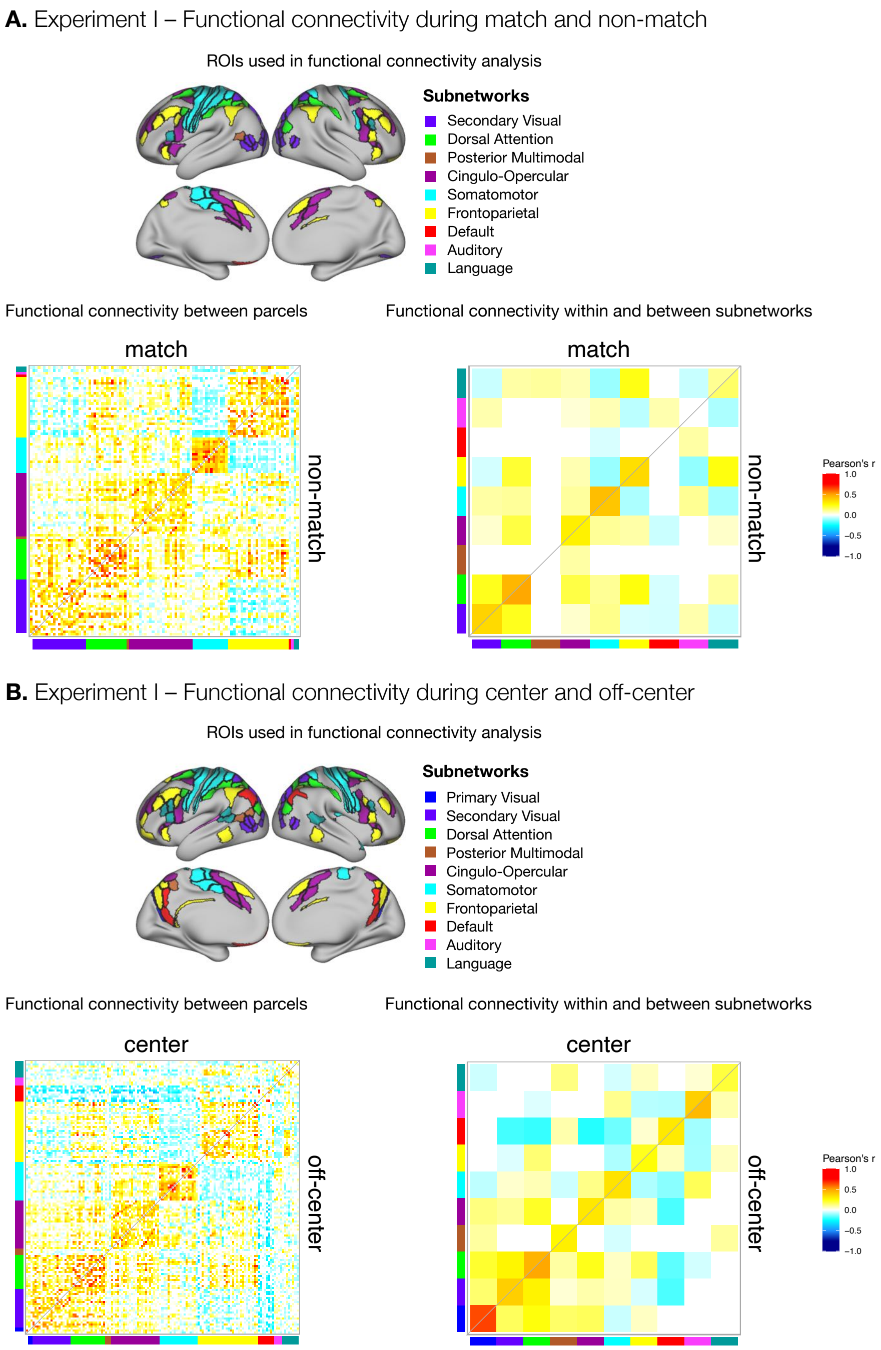

Figure S5. Functional connectivity within the spatial working memory network during the delay period for each task condition in Experiment I. The figure shows the functional connectivity between **A.** *match* and *non-match* conditions, and **B.** *center* and *off-center* conditions in the Experiment I. ROIs were identified based on activation analyses as any region that was active during the delay period of any of the task conditions or showed differences in delay activity between the same task conditions. ROIs were then assigned to subnetworks as described in Ji et al. (2019). As a measure of functional connectivity, we calculated Pearson's correlation coefficients between the delay activity of individual ROIs or specific subnetworks. Statistically significant correlations were identified using permutation analysis, using FDR to control for multiple comparisons and thresholding the results at $q < 0.05$. The resulting correlation matrices are not symmetric, as they show Pearson's correlation coefficients for the *center* condition in the upper triangle and for the *off-center* condition in the lower triangle.

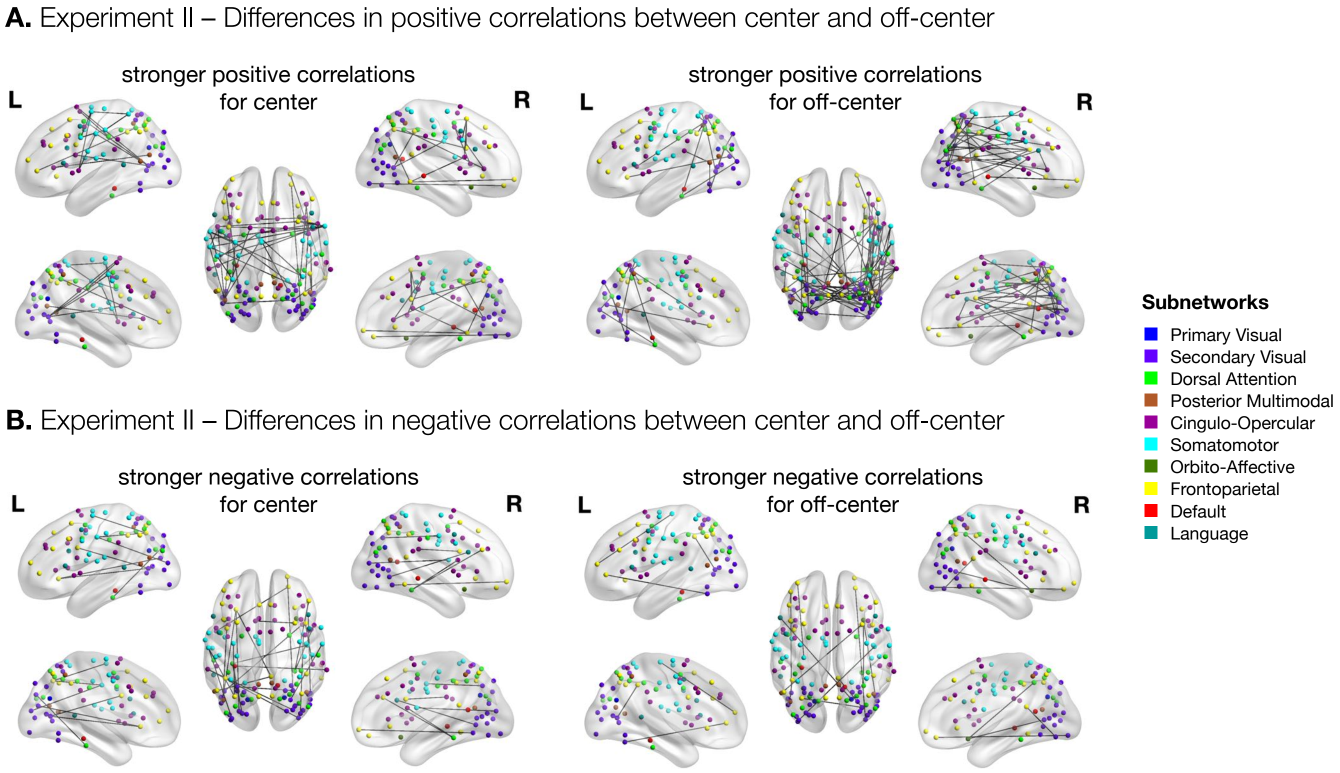

Figure S6. Differences in functional connectivity between individual ROIs during *center* and *off-center* conditions in Experiment II. A. Positive correlations that were stronger for either the *center* or *off-center* condition. **B.** Negative correlations that were stronger for either the *center* or *off-center* condition. As a measure of functional connectivity, we calculated Pearson's correlation coefficients between the delay activity averaged within each ROI. Statistically significant correlations were identified using permutation analysis, using FDR to control for multiple comparisons and thresholding the results at $q < 0.05$.

3 SUPPLEMENTARY TABLES

Note: We only identified peaks from regions with an area of at least 30 mm^2 or a volume of at least 35 voxels. We used a spatial smoothing of 10 FWHM before identifying the peaks. Regions were categorized according to the HCP-MMP1.0 parcellation (Glasser et al., 2016), where we considered all parcels that collectively covered 70% of the region's surface area.

Table S1. Delay activation peaks across all task conditions and experiments

Contrast (two-tailed *t*-contrast)	Region	Hemisphere	MNI coordinates			Peak value (Z-score)	uncorrected *p*-value	Size (voxels / grayordinates)	Area (mm^2)
			x	y	z				
activations	SCEF, 24dd, p32pr	left	-6.4	13.9	47.8	3.4	< .001	329	224.5
	24dd	left	-10.0	-21.5	45.4	3.2	< .001	46	37.6
	4	left	-29.9	-24.4	61.1	3.5	< .001	84	86.1
	4	left	-28.0	-24.3	61.8	3.4	< .001	61	63.5
	6a, 6d, FEF	left	-23.7	-7.4	50.5	3.6	< .001	814	520.1
	1	left	-31.4	-36.3	67.1	3.1	.003	35	30.4
	3b, 1	left	-36.2	-28.7	51.0	3.5	< .001	470	345.2
	AVI, FOP5	left	-29.6	24.0	5.3	3.3	< .001	134	112.4
	FOP4, 44	left	-40.8	18.9	6.4	3.1	.004	46	37.7
	V4	left	-23.5	-86.4	18.4	2.8	.010	36	30.9
	IPS1, IP0, V3B	left	-27.4	-71.5	25.6	3.6	< .001	303	231.8
	AIP, IP2, 2, LIPd	left	-32.9	-50.9	53.6	3.5	< .001	848	300.3
	7PC, LIPv	left	-32.6	-51.8	54.9	3.5	< .001	79	37.3
	VIP, MIP, 7Pl, LIPv, 7Am	left	-24.8	-58.4	56.8	3.5	< .001	561	207.5
	MST, MT	left	-43.1	-66.8	6.3	3.2	< .001	108	48.0
	6r	left	-54.8	6.6	18.0	3.2	< .001	61	50.8
	PEF, 6v, 6r	left	-50.6	1.5	36.9	3.5	< .001	207	151.8
	p9-46v	left	-41.5	28.3	32.6	3.0	.005	34	30.6
	SCEF, 8BM	right	7.3	10.7	52.1	3.4	< .001	185	137.8
	6a, FEF	right	27.0	-4.4	48.9	3.6	< .001	528	338.0
	AIP, IP2	right	38.9	-39.7	42.0	3.5	< .001	355	111.0
	FOP5, FOP4	right	32.6	24.5	9.5	3.4	< .001	138	106.7
	MIP, VIP, 7Am, 7Pl	right	22.1	-64.7	55.1	3.5	< .001	429	181.5
	IPS1, IP0, V3B	right	30.2	-67.9	29.9	3.5	< .001	226	167.3
	AIP, LIPv	right	35.1	-46.0	51.4	3.5	< .001	356	121.7
	MST, MT	right	44.8	-64.2	6.4	3.2	< .001	92	47.1
	44, FOP4	right	46.2	15.3	1.5	3.0	.002	44	39.2
	PEF, 6v, 6r	right	48.6	4.2	32.7	3.4	< .001	196	134.2
	cerebellum	right	12.0	-56.0	-46.0	3.1	< .001	40	–

Table S2. Delay deactivation peaks across all task conditions and experiments

Contrast (two-tailed *t*-contrast)	Region	Hemisphere	MNI coordinates			Peak value (Z-score)	uncorrected *p*-value	Size (voxels / grayordinates)	Area (mm²)
			x	y	z				
deactivations	POS1, ProS, RSC	left	-10.4	-52.0	5.1	-3.4	< .001	293	192.1
	d23ab, 31pv	left	-3.4	-40.9	32.7	-3.2	< .001	96	75.6
	7m, POS2	left	-2.2	-65.8	27.5	-3.2	< .001	51	63.6
	31pd, 31pv	left	-10.5	-52.7	30.5	-3.3	.001	186	125.7
	43, OP4	left	-60.4	-4.8	12.6	-2.9	.009	90	66.2
	PHA3, VVC	left	-32.5	-32.0	-16.2	-3.2	< .001	101	111.4
	V1	left	-2.6	-70.5	4.8	-3.1	< .001	63	79.8
	V1, V3, V2	left	-2.4	-88.7	16.8	-3.2	< .001	267	274.2
	V1	left	-9.8	-57.1	1.8	-3.1	< .001	38	49.2
	POS1	left	-3.6	-61.6	16.3	-3.2	.002	56	65.8
	v23ab, d23ab, RSC	left	-7.0	-51.6	27.3	-3.3	.001	100	84.7
	PHA1, PHA2, VMV2	left	-21.4	-40.0	-12.0	-3.3	< .001	182	162.1
	8Ad	left	-19.7	27.8	48.8	-2.9	.003	42	39.6
	cerebellum	left	-20.0	-66.0	-16.0	-2.8	.003	43	–
	cerebellum	left	-20.0	-48.0	-54.0	-2.9	.003	47	–
	cerebellum	left	-28.0	-80.0	-38.0	-3.1	< .001	511	–
	cerebellum	left	-30.0	-36.0	-26.0	-3.0	< .001	47	–
	cerebellum	left	-40.0	-82.0	-36.0	-3.1	.009	67	–
	31pd, v23ab, 31pv, d23ab, RSC	right	3.5	-46.7	21.8	-3.4	< .001	476	358.8
	ProS, POS1, RSC	right	7.2	-45.6	7.9	-3.3	.006	140	94.2
	PHA1, VMV1	right	19.2	-40.8	-11.2	-3.2	< .001	124	111.9
	2, 7AL	right	20.8	-40.4	64.2	-3.1	< .001	75	33.6
	PGi	right	51.8	-58.9	26.4	-3.0	.002	134	60.2
	OP2-3, Ig	right	40.8	-12.2	19.9	-3.2	< .001	105	64.5
	OP4, 43	right	51.1	-6.1	9.4	-3.2	< .001	105	68.4
	PHA2, PHA3	right	34.1	-29.8	-17.3	-3.0	.002	37	37.9
	V1, V2	right	4.8	-66.3	5.2	-3.2	< .001	81	102.7
	V1, V2	right	5.2	-87.2	13.1	-3.3	< .001	137	163.8
	V3, V6	right	7.4	-82.9	34.5	-3.2	< .001	143	150.9
	ProS, DVT, V2	right	25.8	-57.1	6.3	-3.2	.005	43	37.5
	cerebellum	right	28.0	-86.0	-44.0	-2.8	.006	56	–
	cerebellum	right	22.0	-76.0	-32.0	-2.9	.003	175	–

Table S3. Match vs. non-match delay activity peak differences in Experiment I

Contrast (two-tailed *t*-contrast)	Region	Hemisphere	MNI coordinates			Peak value (Z-score)	Cluster-wise FWE corrected *p*-value	Size (voxels / grayordinates)	Area (mm^2)
			x	y	z				
match > non-match									
delay	6d	left	-38.8	-12.9	63.5	3.6	.037	35	55.5
	6mp, 6d	left	-19.4	-18.9	72.7	3.5	.037	38	33.9
	cerebellum	right	22.0	-54.0	-24.0	3.9	.002	154	–
	cerebellum	right	10.0	-56.0	-14.0	3.7	.002	51	–
early delay	6d, 4	left	-39.0	-17.4	62.7	3.9	.003	82	98.9
	2, 1	left	-42.4	-33.1	50.0	3.5	.013	102	65.0
	cerebellum	right	22.0	-54.0	-22.0	3.8	.022	78	–
match < non-match									
delay	7Pl, MIP, VIP	left	-14.2	-66.7	57.0	-3.7	.026	178	87.9
	6a	right	25.8	-3.2	49.9	-3.7	.041	90	60.1
	3b	right	54.9	-11.9	47.4	-3.6	.030	70	60.6
	MIP, VIP, 7Pl	right	14.5	-65.5	58.9	-3.7	.030	210	96.3
	3a, 4	right	53.8	-3.5	24.0	-3.6	.030	88	59.5
	cerebellum	right	36.0	-74.0	-28.0	-3.6	.029	80	–
late delay	7Pm, 7Am, 7Pl	left	-11.4	-61.0	58.9	-3.6	.013	121	56.7
	PCV, 7Pm	right	7.3	-62.1	50.2	-3.7	.029	113	58.8
	cerebellum	right	30.0	-72.0	-28.0	-3.5	.015	72	–

Table S4. Center vs. off-center delay activity peak differences in Experiment I

Contrast (two-tailed *t*-contrast)	Region	Hemisphere	MNI coordinates			Peak value (Z-score)	Cluster-wise FWE corrected *p*-value	Size (voxels / grayordinates)	Area (mm²)
			x	y	z				
center > off-center									
delay	4	left	-39.2	-14.8	56.2	3.8	.006	63	67.0
	6d	left	-34.8	-10.0	63.1	3.7	.006	56	67.0
	2, 1	left	-47.8	-27.6	51.3	3.6	.011	90	61.5
	2, 1	left	-54.9	-18.6	49.2	3.6	.011	63	40.3
	cerebellum	right	30.0	-48.0	-24.0	4.0	.002	316	–
early delay	6d	left	-30.6	-12.5	63.9	4.1	.002	147	145.6
late delay	cerebellum	right	16.0	-52.0	-20.0	3.4	.012	60	–
center < off-center									
delay	7Pm, PCV, 7Pl, 7m, MIP	left	-5.9	-58.4	44.3	-4.3	.002	399	197.9
	IP1, PGs	left	-38.3	-67.2	46.7	-3.6	.013	170	94.5
	3b, 3a	left	-52.4	-12.1	31.5	-3.8	.007	152	116.2
	4, 3a	right	35.9	-22.3	56.5	-3.5	< .001	65	50.4
	4	right	25.3	-23.2	65.8	-3.6	< .001	54	45.7
	3a, 4	right	8.3	-27.8	73.9	-3.4	< .001	63	53.3
	3b, 3a	right	30.2	-28.6	64.3	-3.9	< .001	208	163.5
	3b	right	48.0	-15.0	41.0	-4.0	.004	110	90.8
	7Pm, 7Pl, PCV	right	20.8	-72.1	48.6	-3.6	.026	160	83.0
	3b, 3a	right	55.9	-7.8	28.4	-3.5	.004	94	81.3
early delay	3a, 3b	right	40.9	-24.4	53.6	-3.6	.002	116	80.3
late delay	6a, i6-8	left	-24.4	4.4	53.0	-4.1	.017	224	135.7
	PFm, IP2	left	-48.9	-39.8	45.9	-4.0	.021	165	75.5
	7Am, 7Pm, 7Pl, VIP, PCV	left	-9.3	-64.7	57.6	-4.4	< .001	543	235.2
	IP1, MIP	left	-25.3	-65.0	38.2	-3.9	< .001	230	108.2
	LIPd, IP2	left	-34.4	-50.5	39.4	-3.9	.021	122	49.1
	PGp, IP0, V3B	left	-35.5	-85.3	27.2	-3.9	< .001	187	149.5
	PGs, PGp, IP0	left	-36.7	-77.8	36.4	-3.9	< .001	110	92.0
	3b	left	-51.3	-11.4	27.3	-3.5	.046	27	32.5
	3a, 3b	left	-49.0	-11.1	26.8	-3.5	.046	69	51.3
	V2	left	-10.1	-82.1	-11.1	-3.9	.028	44	46.7
	V1	left	-3.8	-87.7	-3.6	-3.9	.028	56	77.5
	cerebellum	left	-8.0	-76.0	-30.0	-3.8	.005	75	–
	7Pm, 7Pl, MIP, POS2	right	7.8	-64.1	51.8	-4.0	.005	341	146.8
	IP1, MIP, IP0	right	30.7	-64.0	34.1	-3.9	.001	144	79.0
	IPS1, MIP	right	28.3	-64.0	35.1	-3.9	.001	41	32.7
	PGp, PGs, IP0	right	41.2	-76.0	33.1	-4.2	.001	201	145.3
	PFm, IP2	right	50.9	-34.9	47.7	-3.6	.031	202	65.4
	3b	right	60.7	-6.2	32.6	-3.7	.018	74	61.2
	3a, 4	right	44.4	-10.1	30.8	-3.5	.018	38	33.0
	6a, i6-8	right	28.9	9.9	52.9	-3.9	.026	117	75.3

Table S5. Center vs. off-center delay activity peak differences in Experiment II

Contrast (two-tailed *t*-contrast)	Region	Hemisphere	MNI coordinates x	y	z	Peak value (Z-score)	Cluster-wise FWE corrected *p*-value	Size (voxels / grayordinates)	Area (mm²)
center > off-center									
delay	6d, 4	left	-38.8	-9.6	62.0	4.2	.002	171	177.8
	1, 2	left	-54.0	-21.5	45.7	4.3	.002	262	159.2
	OP1, OP4, PF	left	-50.8	-23.6	19.1	3.9	.004	230	136.7
	cerebellum	right	26.0	-46.0	-28.0	4.0	.006	146	–
	cerebellum	right	22.0	-54.0	-16.0	4.0	.006	138	–
early delay	SCEF	left	-8.9	-5.4	67.6	4.3	.047	64	44.1
	6d, 4, 6mp	left	-34.8	-11.2	64.2	4.9	.006	375	307.0
	2	left	-39.3	-34.3	46.5	4.0	.006	136	65.8
	1, 2	left	-52.2	-23.2	45.5	4.1	.006	261	159.9
	3b, 3a	left	-38.8	-24.3	46.9	3.9	.006	120	89.3
	PF, PFt	left	-54.2	-26.3	35.6	3.8	.006	84	53.8
	cerebellum	right	10.0	-52.0	-20.0	3.9	< .001	506	–
center < off-center									
delay	6a	left	-22.1	-5.4	56.6	-4.0	.004	221	139.0
	IPS1, DVT, V7	left	-20.6	-68.6	42.5	-3.8	.001	154	132.6
	PCV, 7Am	left	-5.7	-55.8	55.6	-3.7	.040	99	52.7
	VIP, 7Am, LIPv	left	-22.0	-60.2	59.5	-4.2	.001	234	95.5
	PGp, PGs	left	-34.7	-84.2	30.5	-3.8	.019	88	82.8
	4, 3a	right	30.7	-23.3	52.4	-3.7	.024	59	47.3
	6a	right	24.8	-3.4	50.8	-4.4	.006	219	129.5
	IPS1, MIP, 7Pl, VIP	right	24.5	-70.2	44.5	-4.4	.006	321	212.6
	7Am, VIP	right	13.2	-61.1	61.6	-3.8	.006	81	39.2
	7Am, PCV	right	5.5	-47.7	56.0	-4.0	.006	173	78.4
	IP0, PGs	right	36.8	-71.3	39.2	-3.8	.018	113	92.0
	LIPv, AIP	right	27.2	-62.6	51.5	-4.0	.006	196	82.3
	TPOJ3, PGp	right	47.3	-70.3	16.1	-3.7	.036	50	33.8
	POS1	right	18.4	-61.4	26.7	-3.5	.024	48	45.9
late delay	6a	left	-26.0	-5.0	49.0	-4.4	< .001	262	161.2
	DVT, POS2	left	-11.8	-73.1	41.2	-3.9	< .001	98	64.6
	PCV	left	-5.8	-54.2	51.5	-3.8	.008	122	66.0
	VIP, 7Pm, 7Am	left	-21.9	-62.0	56.3	-4.0	< .001	242	107.2
	PGp, PGs	left	-41.1	-76.2	29.6	-3.9	.010	74	72.6
	PCV	right	7.2	-47.1	57.1	-4.0	.001	121	68.3
	6a	right	26.0	-1.4	51.0	-4.2	< .001	168	108.2
	6a	right	23.1	0.2	59.5	-3.7	< .001	48	35.3
	IPS1, MIP, 7Pl	right	23.1	-67.9	45.9	-4.2	< .001	237	145.4
	7Am, 7Pm	right	11.7	-62.0	60.3	-4.0	.001	136	58.6

Table S6. Center vs. off-center parcellated delay activity differences in Experiment I

Contrast (two-tailed *t*-contrast)	Region	Hemisphere	Z-score	FDR corrected *p*-value
center > off-center				
delay	6d	left	3.4	.030
	FOP2	left	3.2	.036
center < off-center				
delay	RSC	left	-3.1	.036
	POS2	left	-2.9	.036
	PCV	left	-4.2	.030
	STV	left	-3.2	.030
	7Pm	left	-4.3	.030
	7m	left	-4.3	.030
	POS1	left	-3.3	.031
	7PL	left	-3.7	.030
	MIP	left	-3.3	.036
	a47r	left	-3.1	.030
	IFSp	left	-3.3	.032
	10pp	left	-3.1	.030
	i68	left	-3.5	.036
	PBelt	left	-3.7	.036
	TE1p	left	-3.3	.030
	TPOJ1	left	-3.0	.030
	TPOJ3	left	-2.9	.043
	DVT	left	-3.5	.040
	PGp	left	-3.5	.030
	IP2	left	-3.1	.035
	IP1	left	-3.3	.036
	PGs	left	-3.3	.031
	A4	left	-3.5	.036
	4	right	-3.4	.030
	3b	right	-4.0	.030
	POS2	right	-2.8	.030
	7Pm	right	-3.6	.036
	7m	right	-2.6	.030
	POS1	right	-2.8	.036
	7PL	right	-3.6	.036
	1	right	-2.7	.037
	3a	right	-3.8	.030
	OP23	right	-3.0	.036
	STGa	right	-2.3	.045
	TE1p	right	-2.8	.030
	TPOJ1	right	-2.3	.036
	DVT	right	-2.7	.030
	PGp	right	-3.2	.030
	IP1	right	-2.9	.044
	IP0	right	-3.1	.030
	PGs	right	-3.7	.030
	MBelt	right	-3.3	.030

Table S7. Center vs. off-center parcellated delay activity differences in Experiment II – Part 1

Contrast (two-tailed *t*-contrast)	Region	Hemisphere	Z-score	FDR corrected *p*-value
center > off-center				
delay	24dd	left	2.5	.040
	1	left	4.0	.006
	2	left	3.3	.005
	6d	left	4.5	.002
	6mp	left	2.6	.024
	OP4	left	3.5	.002
	OP1	left	3.8	.003
	PFcm	left	3.5	.006
	FOP1	left	3.7	.006
	FOP2	left	2.8	.024
	PFt	left	2.7	.037
	PF	left	3.7	.012
	PFcm	right	3.1	.016
	FOP1	right	2.5	.035
	FOP2	right	3.0	.009

Table S8. Center vs. off-center parcellated delay activity differences in Experiment II – Part 2

Contrast (two-tailed *t*-contrast)	Region	Hemisphere	Z-score	FDR corrected *p*-value
center < off-center				
delay	FEF	left	-2.6	.035
	V7	left	-3.5	< .001
	IPS1	left	-4.6	.006
	FFC	left	-2.5	.041
	V3B	left	-3.7	.002
	PCV	left	-4.3	.004
	7Pm	left	-3.4	.006
	7Am	left	-4.1	< .001
	7PL	left	-4.0	.002
	LIPv	left	-3.7	.009
	VIP	left	-5.7	.003
	MIP	left	-3.8	.009
	LIPd	left	-2.9	.029
	6a	left	-4.6	.003
	PHA3	left	-3.0	.012
	TPOJ3	left	-4.0	.003
	DVT	left	-3.4	.007
	PGp	left	-4.9	.002
	IP0	left	-3.0	.012
	V6A	left	-3.0	.020
	VMV3	left	-2.9	.013
	PHA2	left	-2.6	.041

Table S9. Center vs. off-center parcellated delay activity differences in Experiment II – Part 3

Contrast (two-tailed *t*-contrast)	Region	Hemisphere	Z-score	FDR corrected *p*-value
center < off-center				
delay	V8	right	-3.0	.017
	4	right	-2.5	.013
	3b	right	-2.7	.030
	RSC	right	-2.5	.030
	V7	right	-4.2	.002
	IPS1	right	-5.1	.002
	V3B	right	-3.2	.009
	MT	right	-3.2	.009
	PCV	right	-4.1	.004
	7Pm	right	-3.5	< .001
	POS1	right	-2.7	.017
	7AL	right	-2.4	.047
	7Am	right	-4.2	< .001
	7PL	right	-3.7	< .001
	LIPv	right	-4.8	.004
	VIP	right	-4.8	.004
	MIP	right	-4.5	.004
	1	right	-2.3	.042
	3a	right	-2.5	.030
	IFJp	right	-2.3	.039
	a10p	right	-2.7	.030
	LIPd	right	-2.6	.048
	6a	right	-5.0	.004
	i68	right	-3.2	< .001
	AAIC	right	-2.6	.018
	AIP	right	-3.1	.012
	PHA3	right	-2.6	.030
	STSvp	right	-2.5	.030
	TE1p	right	-3.0	.008
	TPOJ3	right	-4.0	.006
	DVT	right	-3.2	.015
	PGp	right	-4.4	.006
	IP1	right	-2.8	.030
	IP0	right	-4.4	.004
	V6A	right	-4.2	.002
	VMV3	right	-2.6	.025
	V4t	right	-3.0	.028
	FST	right	-2.9	.024
	V3CD	right	-3.1	.012
	LO3	right	-3.0	.009

REFERENCES

Allen, P. J., Josephs, O., and Turner, R. (2000). A Method for Removing Imaging Artifact from Continuous EEG Recorded during Functional MRI. *NeuroImage* 12, 230–239. doi:10/dqfx4j

Funahashi, S. (2015). Functions of delay-period activity in the prefrontal cortex and mnemonic scotomas revisited. *Front. Syst. Neurosci.* 9, 2. doi:10.3389/fnsys.2015.00002

Glasser, M. F., Coalson, T. S., Robinson, E. C., Hacker, C. D., Harwell, J., Yacoub, E., et al. (2016). A multi-modal parcellation of human cerebral cortex. *Nature* 536, 171–178. doi:10/f8z3gb

Ji, J. L., Spronk, M., Kulkarni, K., Repovš, G., Anticevic, A., and Cole, M. W. (2019). Mapping the human brain's cortical-subcortical functional network organization. *NeuroImage* 185, 35–57. doi:10/ggd8dm

Jia, Y. and Tyler, C. W. (2019). Measurement of saccadic eye movements by electrooculography for simultaneous EEG recording. *Behav Res* 51, 2139–2151. doi:10/ghvmpr

Mullen, T. (2012). NTRC: CleanLine.

Niazy, R., Beckmann, C., Iannetti, G., Brady, J., and Smith, S. (2005). Removal of FMRI environment artifacts from EEG data using optimal basis sets. *NeuroImage* 28, 720–737. doi:10.1016/j.neuroimage.2005.06.067

van der Meer, J. N., Pampel, A., Van Someren, E. J., Ramautar, J. R., van der Werf, Y. D., Gomez-Herrero, G., et al. (2016). Carbon-wire loop based artifact correction outperforms post-processing EEG/fMRI corrections—A validation of a real-time simultaneous EEG/fMRI correction method. *NeuroImage* 125, 880–894. doi:10.1016/j.neuroimage.2015.10.064
